# Using Wearables in Mental Health Care for Children and Adolescents: A Scoping Review

**DOI:** 10.1007/s10802-025-01408-9

**Published:** 2026-01-26

**Authors:** Catharina E. Bergwerff, Renate S. M. Buisman, Nikki Nibbering, Siri D. S. Noordermeer

**Affiliations:** 1 Institute of Education and Child Studies, Leiden University, Leiden, The Netherlands; 2 Department of Clinical Neuro- and Development Psychology, Vrije Universiteit Amsterdam, Amsterdam, Netherlands

**Keywords:** Wearable technology, Paediatric mental health, Physiology, Externalizing behaviour, Forensic youth care, Assessment

## Abstract

Wearable technologies offer promising opportunities for non-invasive, continuous monitoring of physiological processes relevant to paediatric mental health. Although their use in adult psychiatry is growing, little is known about their application in mental health care for youth. This scoping review maps the current state of research on wearable technologies used to assess or support mental health in children and adolescents, with special attention to externalizing behaviours and forensic or residential settings. We systematically searched four databases (PubMed, PsychINFO, Web of Science, Embase) for peer-reviewed English-language studies involving participants aged 0 to 17, using wearable devices to assess physiological markers related to mental health. A total of 85 studies were included. Most studies focused on monitoring sleep (54) using wrist-worn actigraphy, particularly in youth with attention-deficit/hyperactivity disorder, autism spectrum disorder, or internalizing problems. A smaller number examined autonomic nervous system responses (19), motor activity (8), or brain activity and eye gazing (5). Of all 85 studies, 10 focused on externalizing behaviours or youth in forensic or residential care. Although feasibility was generally good, wearables were almost exclusively used for assessment, not intervention. Wearables are increasingly used to assess physiological parameters linked to mental health symptoms in youth. However, there is a lack of clinical implementation studies, particularly in high-risk populations. Future research should move beyond feasibility and direct assessment, and explore wearable-based interventions and just-in-time feedback strategies in clinical and forensic youth care.

**Supplementary Information:**

The online version contains supplementary material available at 10.1007/s10802-025-01408-9.

In recent years, technological advancements have revolutionized various fields, with wearable devices - or wearables for short - emerging as promising tools in the health care and behavioural sciences sector (Roos & Slavich, 2023). In health and behavioural research, wearables refer to electronic devices that can be worn on the body, often resembling everyday accessories such as watches or wristbands, to track an individual’s physiological activity (Kazanskiy et al., 2024). These devices offer a unique window into the body’s stress regulation systems by tracking metrics like heart rate variability, skin conductance, sleep quality, and physical activity (Roos & Slavich, 2023). Such physiological indicators are particularly valuable for understanding stress dysregulation and identifying patterns associated with behavioural escalation, for instance in juvenile forensic populations. Wearables can detect subtle shifts in autonomic nervous system (ANS) activity that may precede emotional or behavioural dysregulation, before it manifests outwardly (Hickey et al., 2021). This is especially relevant in youth populations, where difficulties in recognizing or articulating internal states can hinder timely intervention (Rakoczy, 2022). The integration of wearables with Ecological Momentary Assessment (EMA) adds further value, allowing researchers and clinicians to examine real-time correlations between physiological responses, emotional states, and contextual triggers (Tutunji et al., 2023). In this way, wearables may support the early identification of risk states and promote timely and targeted support strategies (e.g., Hickey et al., 2021; Hilty et al., 2021; Long et al., 2022).

Although most wearable-based research has focused on adult populations, there is a growing interest in their application within child and adolescent mental health settings (Sequeira et al., 2020; Welch et al., 2022). A recent scoping review revealed fewer than 20 studies in child and adolescent psychiatric samples that gathered data through wearables, many of which were feasibility trials using electrocardiogram (ECG) chest straps or wrist-worn sensors (Welch et al., 2022). Those studies show that wearable technology holds potential for identifying autonomic patterns associated with psychiatric conditions such as autism spectrum disorder (ASD), anxiety, and mood disorders. Nevertheless, significant gaps remain in the evidence base, particularly regarding larger-scale trials and applications in forensic youth populations (Welch et al., 2022). Furthermore, while Welch et al. (2022) provided a valuable foundation by mapping the use of wearable devices in child and adolescent psychiatry, the field has evolved considerably in a short period, warranting an updated synthesis of the literature.

Collecting objective psychophysiological data with wearables in young children and adolescents may be of particular importance, since young children often struggle to accurately label and articulate their experienced stress and emotions (Rakoczy, 2022). Self-reported data obtained by children can therefore be less reliable, whereas wearables may provide a more accurate representation of their physiological state. Further, monitoring a child’s psychophysiological data may allow for the early detection of dysregulation associated with mental health conditions, before symptoms become apparent. This may enhance the timeliness and accuracy of diagnostic assessment, enabling mental healthcare providers to address potential issues proactively. Given the impact deviant development can have on adult-life outcomes, including delinquent behaviours and offending, this is crucial. Fortunately, wearables have gained attention in recent years for their potential in assessing mental health conditions among youth.

Furthermore, wearable technology has opened new avenues for mental health interventions. Wearable devices can facilitate biofeedback, enhance patient engagement, and promote long-term behaviour change through personalized feedback and reminders (Hilty et al., 2021). This can be of help for children and adolescents with a variety of mental health issues. For instance, it has been suggested that children with ASD may benefit from wearables that incorporate sensors such as speakers, microphones, and heart rate monitors to enhance communication and interaction through visual signals (Koumpouros & Kafazis, 2019). Further, wearables may aid children with internalizing problems, by detecting increasing physiological signals of stress (including increased heart rate) and prompting them to apply relaxation techniques, such as taking deep breaths, listening to music or change activities when feeling overwhelmed (González Ramírez et al., 2023). The use of wearable technology would thereby provide youth with more autonomy to regulate their emotions and behaviour.

The benefits of wearable technology may extend to a large variety of populations, including youth with externalizing problems. A scoping review on digital health interventions for mental health, substance use, and co-occurring disorders in the criminal justice population suggested that wearables hold promise for preventing relapse and aiding in the reintegration of youth from forensic settings (Leach et al., 2022). As wearables may detect substance use, stress or other factors that place individuals at high risk for externalizing behaviours, this technology may provide an opportunity for just-in-time adaptive interventions. The integration of wearable-based interventions in general mental health care, and specifically externalizing behaviours, could thus represent a significant step forward in improving outcomes for young individuals with mental health challenges.

For youth within the juvenile justice system, the potential utility of wearables is multifaceted. These adolescents often deal with complex issues such as trauma, impulse control problems, and difficulties with emotion regulation. These factors may elevate the risk of behavioural escalation and recidivism (Ryan et al., 2014). Wearables could enable continuous stress monitoring and offer real-time feedback to identify rising arousal levels. This would allow for timely interventions, including biofeedback and self-regulation techniques. Moreover, in aftercare or reintegration phases, wearables may help prevent relapse through just-in-time adaptive interventions (de Looff et al., 2024). The potential of wearable technology to support behaviour regulation, emotional awareness, and stabilization suggests that its use in forensic youth care deserves further exploration. Beyond diagnostics, wearables may empower youth to engage with their physiological states and autonomously regulate behaviour, which may help reduce the risk of reoffending. However, it is important to know how accurate assessment is before being able to implement this.

Given the burgeoning interest in this field, a comprehensive review of the current landscape of wearables in paediatric mental health care is essential for not only understanding their potential, but also for identifying gaps in research, and guiding future studies and innovations on wearables in mental health care. The current scoping review aims to explore the existing literature on the use of wearables for assessment and treatment purposes in mental health settings in children and adolescents. The current review extends the work of Welch et al. (2022) by focusing not only on the general applicability of wearables in paediatric mental health care, but also by emphasizing the clinical translation of wearable-based monitoring, with a particular interest in youth with externalizing behaviours and those in forensic or residential settings. This is a population with distinct challenges and care needs, where real-time physiological data may help detect early warning signs and support intervention. As such, this review not only updates the evidence base but also aims to identify practical implications for mental health and forensic youth care.

The aims of this scoping review are, therefore, threefold. First, to provide a comprehensive overview of studies using wearable technologies for the assessment (and where applicable, the intervention) of mental health problems in children and adolescents. Second, to summarize the main findings of these studies in relation to physiological parameters such as heart rate variability, sleep, and activity levels, and how these are described in connection with youth mental health problems. Third, to review the reported feasibility of using wearable technologies in these populations. In addition to these core aims, the review specifically maps studies focusing on externalizing behaviours and youth in residential or forensic settings, with the goal of identifying implications for forensic youth care and rehabilitation. Finally, the review explores how wearable-based interventions are currently being applied, if at all, to support mental health treatment, for instance through relapse prevention, just-in-time feedback, engagement enhancement, or reintegration support following forensic placement.

This study does not seek to quantify prevalence or effect sizes, nor to establish causal relationships. Instead, in line with the scoping review methodology, it aims to map the breadth of existing literature, describe patterns in current applications of wearables, and identify gaps and opportunities for future research, clinical practice and forensic care.

## Methods

The current scoping review was conducted in accordance with the Preferred Reporting Items for Systematic reviews and Meta-Analyses extension for Scoping Reviews (PRISMA-ScR) checklist (Tricco et al., 2018). The protocol was registered on the Open Science Framework (https://osf.io/g76cm).

### Eligibility 

Studies were eligible for inclusion if:

1. They were published in peer-reviewed journals in English.
2. They reported primary data (instead of reviews or meta-analyses).
3. They had an appropriate design (i.e., randomized controlled trials, non-randomized experimental studies, cohort studies and case-control studies).
4. Participants were 17 years or younger. The main reason for this strict age-criterion is that often the mental health system has a clear division between youth and adult populations.
5. Wearable technology was used, according to the definition of the concept ‘wearable’ used in this paper - electronic devices that can be worn on the body, often resembling everyday accessories such as watches or wristbands, to track an individual’s physiological activity. These devices are equipped with sensors and software designed to collect and visualize physiological data in real-time. Hardware measuring brain activity or eye-tracking should be ambulatory (e.g., not connected to a computer).
6. Given the focus on clinical populations, the wearable data should have been studied in relation to the presence of a mental disorder (as established with a DSM- or ICD-based diagnosis) or, given the importance for the forensic field, in relation to symptoms of externalizing behaviours.

Study exclusion criteria include conference abstracts and dissertations.

### Search Strategy

Four databases were searched (PubMed, PsycINFO, Web of Science and Embase) in August 2024. Keyword searches in these databases consisted of a combination of search terms (youth AND mental health AND technology (wearables). See supplementary materials for a complete overview of the search strategy.

### Selection of Sources

Based on the inclusion criteria, study selection, screening and full-text assessment took place by authors CB and SN. In the first round, screening was based on title and abstract. The second round comprised reviewing selected full-text studies, and subsequent data-extraction. Disagreements between the reviewers were to be resolved by discussion, or otherwise a third person (author RB) would be consulted.

### Data Charting

Data were extracted by authors CB, SN and NN. First, all three authors extracted data from the same articles, to check and confirm agreement. After clear agreement, remaining data were extracted on two levels; on a more general level to generate an overview of all included studies (see Table s1), and on a more specific level for the results on the different outcome domains. The outcome domains were based on the physiological parameters assessed in the included studies (such as sleep parameters or electrodermal activity). The results were synthesized per domain and if possible (i.e. in the case of enough studies) subdivided per disorder (e.g. attention-deficit/hyperactivity disorder (ADHD), ASD, internalizing disorders, externalizing disorders).

This first, general level, of extracted data included for each study; (a) information on the population, specifically recruitment source, age and gender, (b) assessed parameters (sleep, autonomic nervous system, motion and activity, brain activity and eye gazing), and (c) type of wearable. The second, outcome related level of extracted data included (a) sample characteristics, (b) study aim, (c) outcome measures, (d) main findings, and (e) where applicable feasibility.

## Results

### Study Selection

**Fig. 1:**
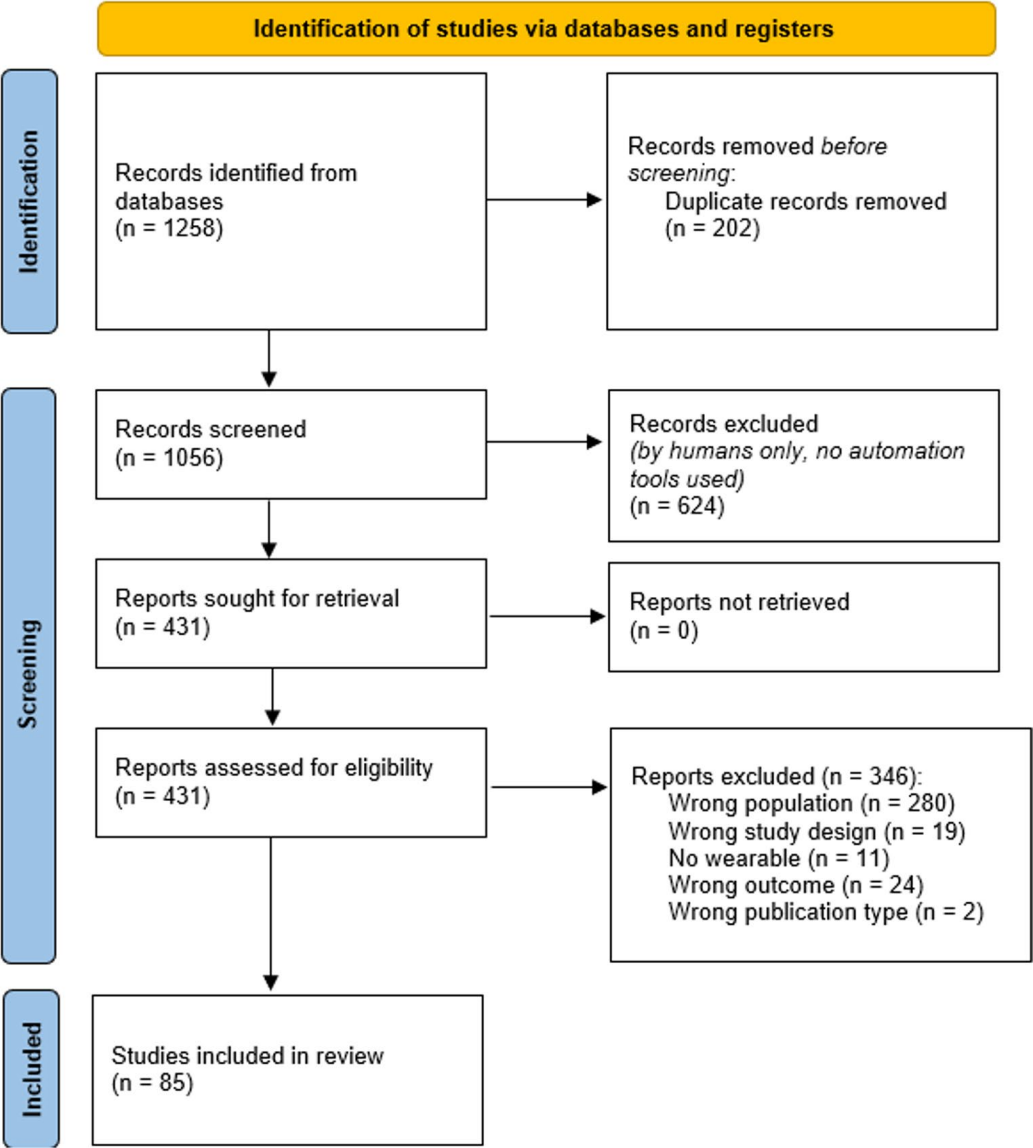
PRISMA-ScR flowchart for study selection

In Fig. 1, a PRISMA-ScR flowchart of the study selection process is presented. The comprehensive systematic search was executed using Rayyan systematic review software (Ouzzani et al., 2016). This tool facilitates organizing citations and enables blind screening of articles among reviewers. The outcomes of the systematic search and screening of abstracts and full texts conducted via Rayyan are illustrated in Fig. 1.

After duplicates were removed, 1,056 studies were included for screening. Manual searches of reference lists of included studies identified no extra studies. In the first round, screening was based on title and abstract, leading to exclusion of 624 studies, since these abstracts did not mention the use of wearable technologies or smart devices for assessing or remediating mental health problems in a paediatric population. After reviewing the 431 full-text studies, 346 studies were excluded. The primary reasons for exclusion, with the accompanying numbers of excluded studies for that reason, are presented in Fig. 1. Disagreements between the reviewers were resolved by discussion, and in all cases led to agreement. The agreement among the reviewers was excellent with kappa = 0.96 for abstract/title screening, and kappa = 0.95 for full text screening. A total of 85 studies met eligibility criteria and were included in this review. A concise summary of the included studies is presented in Table s1.

### Assessment of Sleep

Many of the studies that were included in this review, assessed sleep associated variables in various paediatric conditions. These 54 studies are summarized in Tables 1, 2, 3 and 4. Of these, 22 studies focused on ADHD, 18 on ASD, 9 on internalizing disorders, and 6 on externalizing disorders or behaviours. One of the studies was both part of the studies on ADHD and on internalizing disorders. In all studies, accelerometer devices were used, with the large majority being actigraphy watches.

The primary aim of the 22 studies that included participants with ADHD (see Table 1), was to assess the presence of sleep impairments, primarily at the group level (ADHD versus typically developing [TD]), and in some studies, also at the dimensional level, such as through correlations. Overall, the findings indicate impairments on several sleep parameters for children with ADHD, with 17 studies reporting group differences. Of these, the most consistently reported were impairments in sleep onset latency (Hazari et al., 2015; Langberg et al., 2019; Lee et al., 2014; Moreau et al., 2014; Ziegler et al., 2021), or in sleep efficiency (Hazari et al., 2015; Knight & Dimitriou, 2019; Moreau et al., 2014; Tonetti et al., 2020; Zerón-Rugerio et al., 2021; Ziegler et al., 2021), and a shorter sleep duration (Becker et al., 2019; Kim et al., 2023; Miano et al., 2019; Moreau et al., 2014). Parameters that were less often studied, but where children with ADHD showed deviations from typically developing children, included greater variability or overall nighttime awakenings (Langberg et al., 2019; Lee et al., 2014; Neto & Nunes, 2017), more sleep movements, a higher fragmentation index (Lee et al., 2014; Tonetti et al., 2020), more variability in sleep offset and time in bed (Kim et al., 2023; Langberg et al., 2019; Melegari et al., 2020), and a larger sleep deficit during the day (Ziegler et al., 2021). The other five studies reported no differences on diverse sleep parameters for children with ADHD compared to typically developing children (Bergwerff et al., 2016; Cremone-Caira et al., 2020; Faedda et al., 2016; Fredrick et al., 2022; Sanabra et al., 2021). Assessed parameters in these five studies included sleep efficiency (Cremone-Caira et al., 2020; Faedda et al., 2016; Fredrick et al., 2022; Sanabra et al., 2021), sleep onset time (Cremone-Caira et al., 2020; Fredrick et al., 2022; Sanabra et al., 2021), time in bed (Bergwerff et al., 2016; Fredrick et al., 2022), total sleep time (Bergwerff et al., 2016; Cremone-Caira et al., 2020; Faedda et al., 2016; Fredrick et al., 2022; Sanabra et al., 2021), sleep onset latency (Bergwerff et al., 2016; Faedda et al., 2016; Sanabra et al., 2021), nocturnal activity (Bergwerff et al., 2016; Faedda et al., 2016; Sanabra et al., 2021), wake-up time (Cremone-Caira et al., 2020), wake after sleep onset (Cremone-Caira et al., 2020; Sanabra et al., 2021), morning arising latency (Bergwerff et al., 2016), average wake and sleep bout durations (Bergwerff et al., 2016), bedtime (Cremone-Caira et al., 2020), sleep offset time (Cremone-Caira et al., 2020), and 5 least active hours (Faedda et al., 2016).

**Table 1:** Studies using wearables to assess sleep in children and adolescents – attention-deficit/hyperactivity disorder (ADHD)

Study reference	Sample characteristics	Study aim	Outcome measures	Main findings
Becker et al. (2019)	162 ADHD, 140 TD	Examine and compare sleep patterns of adolescents with and without ADHD.	sleep onset and offset time; time in bed; sleep efficiency; wake after sleep onset	Adolescents with ADHD had shorter sleep duration than adolescents without ADHD.
Bergwerff et al. (2016)	63 ADHD, 61 TD	Gain insight into sleep problems in children with ADHD.	time in bed; total sleep time; nocturnal motor activity; sleep onset latency; morning arising latency; average wake bout duration; and average sleep bout duration	Medication-free children with ADHD have normal sleep quality and quantity
Bundgaard et al. (2018)	24 ADHD, 25 TD	Investigate sleep pattern and night-to-night variability in toddlers with ADHD.	sleep latency total sleep time; number of interruptions of sleep	ADHD traits are associated with an increased night-to-night variability but not a prolonged sleep latency or shorter total sleep time.
Cremone-Caira et al. (2020)	11 ADHD, 15 TD	Determine whether children with ADHD were able to extend over-night sleep duration and if sleep extension would improve inhibitory control in children with ADHD.	bedtime; wake-up time; sleep onset time; total sleep time; wake after sleep onset; sleep efficiency	Children with ADHD were able to successfully extend sleep duration by 48 min, on average (range between 38 and 91 min) when instructed to advance their time in bed by 90 min. The extension of overnight sleep duration improved inhibitory control in children with ADHD
Cusick et al. (2020)	162 ADHD, 140 TD	Assess whether caffeine use is uniquely associated with sleep functioning in adolescents with ADHD compared with adolescents without ADHD.	total sleep time; wake after sleep onset	There were no significant associations between caffeine use in the afternoon or evening and actigraphy-derived total sleep time or wake after sleep onset for either adolescents with or without ADHD
Faedda et al. (2016)	44 ADHD, 42 TD	Ascertain whether objective measures of locomotor activity can potentially aid in the differential diagnosis of paediatric BD	5 least active hours; mean nocturnal activity; percentage nocturnal activity; total sleep time; sleep onset latency; sleep efficiency	No group differences
Fredrick et al. (2022)	140 ADHD, 162 TD	Assess link between sluggish cognitive tempo and sleep.	sleep onset and offset time; time in bed; sleep efficiency	Higher self-reported sluggish cognitive tempo trait scores were associated with shorter sleep duration and later sleep onset
Hazari et al. (2015)	20 ADHD, 20 TD	Assess sleep disturbances in children with ADHD.	time in bed; sleep onset latency; waking after sleep onset; sleep efficiency	Longer sleep onset latency and reduced sleep efficiency.
Neto and Nunes (2017)	42 ADHD + epilepsy, 21 ADHD, 21 TD	Evaluate sleep organization in children and adolescents with ADHD and epilepsy, and to analyse the influence of methylphenidate.	total sleep time; total time in bed; sleep efficiency; sleep latency; number of awakenings after sleep onset	Group with ADHD and ADHD + epilepsy had more night awakenings.
Kim et al. (2023)	79 ADHD, 1011 TD	Develop machine learning method to predict ADHD and sleep problems in children using wearable data.	30-s-related features: duration asleep, duration in-bed sleep, duration deep sleep, duration light sleep, duration REM sleep, duration waking short periods, duration asleep during nap, duration in bed during nap, percentage deep sleep, percentage light sleep, percentage REM sleep, percentage awake, sleep quality. 60-s-related features: duration asleep, during restlessness, duration wake, sleep quality.	ADHD shorter in-bed sleep and shorter light sleep (30-s) and shorter asleep (60-s).
Knight and Dimitriou (2019)	18 ADHD, 20 TD	Investigate the relationship among sleep, ADHD behaviours, and attention in school-age children with and without ADHD.	time in bed; assumed sleep time; actual sleep time; sleep latency; sleep efficiency; fragmentation index	ADHD group showed poorer sleep quality. In TD group poor sleep quality was associated with ADHD symptoms.
Langberg et al. (2019)	162 ADHD, 140 TD	To examine whether adolescents with ADHD have greater intraindividual variability in sleep/wake patterns compared to adolescents without ADHD.	time in bed; sleep onset time; sleep offset time; wake after sleep onset; night awakenings; sleep efficiency	ADHD group had significantly more variability for time in bed, sleep onset, sleep offset, and wake after sleep onset across the entire observation period. Specifically on schooldays the ADHD group had significantly more variability for time in bed, sleep onset, and efficiency.
Lee et al. (2014)	37 ADHD, 32 TD	Examine neurocognitive functions and nocturnal sleep parameters in ADHD.	total sleep time; sleep latency; sleep efficiency; wake after sleep onset; fragmentation index	ADHD group had longer sleep latency, more wake after sleep onset, and a higher fragmentation index
Miano et al. (2019)	30 ADHD, 25 TD	Assess if children with ADHD have chronic sleep deprivation and if they can be classified into specific sleep related phenotypes.	sleep duration; total sleep time; sleep period time; sleep efficiency; sleep latency; number of awakenings; REM latency; wakefulness after sleep onset	Total sleep time lower in ADHD. ADHD symptoms correlated negatively with total sleep time.
Melegari et al. (2020)	25 ADHD, 21 TD	Assess features of sleep in preschoolers with ADHD.	total sleep duration; sleep onset latency; sleep minutes; wake after sleep onset; mean duration wake episode; sleep efficiency; activity mean; activity index; sleep fragmentation index	Children with ADHD showed higher level of nocturnal activity, both mean activity and activity index, and an increased night-to-night variability for sleep minutes and mean wake episodes. There was a positive correlation between attention problems and both mean wake episode and activity mean.
Moreau et al. (2014)	41 ADHD, 41 TD	Characterize sleep of children with ADHD and examine the potential moderating role of psychostimulant treatment and psychiatric comorbidity.	total sleep time; sleep onset time; wake after sleep onset; sleep efficiency; activity counts per minute	ADHD group lower total sleep, longer onset, lower efficiency (more activity).
Sanabra et al. (2021)	60 ADHD, 60 TD	Assess and measure differences between children and adolescents with ADHD and controls through sleep parameters.	sleep onset time; total sleep time; number of awakening; average length of awakenings; sleep efficiency; sleep onset latency	No differences between ADHD and TD group.
Sidol et al. (2023)	43 ADHD	Exame bidirectional relationship of sleep and behaviour in children with ADHD across a range of behavioural domains.	total sleep time; sleep efficiency; wake after sleep onset	Sleep efficiency was positively related to parent-ratings of ADHD symptoms.
Tonetti et al. (2020)	22 ADHD	Assess differences between sleep timing, quality, and quantity in children with ADHD and explore potential effects om prospective memory performance.	sleep timing, quantity and quality derived from bedtime; get-up time; time in bed; midpoint of sleep; sleep onset latency; sleep onset; total sleep time; wake after sleep onset; mean activity score; wake bouts	Lower sleep efficiency, as well as higher mean activity score, and fragmentation index in ADHD.
Waldon et al. (2016)	24 ADHD, 24 TD	Examine if correlations between actigraphy-derived estimated sleep variables and the same variables based on polysomnography differ between children with and without ADHD.	sleep efficiency; sleep duration; sleep-onset latency	Actigraphy provides a reliable estimate of polysomnography-defined sleep duration in children with ADHD on and off medication, as well as in TD children, but provides a considerably less reliable measure of sleep-onset latency and sleep efficiency, particularly in children with ADHD who are not taking medication
Zerón-Rugerio et al. (2021)	60 ADHD, 60 TD	To study possible alterations in circadian rhythms in subjects with ADHD and to establish possible relationships between these circadian alterations, sleep disorders, obesity, and ADHD symptoms	time in bed; time out bed; total sleep time; total time in bed; latency; efficiency; wake after sleep onset; awakenings and average of awakenings	Children with ADHD showed more total sleep problems, more behavioural sleep problems of initiating and maintaining sleep, more sleep-wake transition disorders, and more excessive daytime somnolence. For specifically the combined subtype ADHD, time in bed and time out bed were longer, as well as sleep breathing disorders.
Ziegler et al. (2021)	24 ADHD, 33 TD	Explore sleep problems in children with ADHD compared to their typically developing peers, with a particular focus on intraindividual variability in the sleep variables	sleep onset latency; sleep efficiency; total counts; sleep deficit; total sleep time; sleep onset time	Children with ADHD showed longer sleep onset latency, higher intraindividual variability in sleep onset latency, more movements during sleep, lower sleep efficiency, and a slightly larger sleep deficit on school days compared with free days.

ADHD = attention-deficit/hyperactivity disorder; TD = typically developing

Regarding associations, four out of the five included studies reported that the level of ADHD symptoms was associated with either increased night-to-night variability (Bundgaard et al., 2018), lower total sleep time (Langberg et al., 2019), or lower total sleep efficiency (Moreau et al., 2014). An association between sleep and ADHD symptoms was also reported in a sample of typically developing children, where a higher level of ADHD symptoms was associated with poorer sleep quality. The final study reported no associations between symptoms of ADHD and any sleep parameters (Bergwerff et al., 2016).

Of the 18 studies focusing on ASD (see Table 2), again, most studies aimed to assess impairments in sleep characteristics on a group level, and some used a dimensional approach. Children with ASD had less minutes of sleep per night (Benson et al., 2023; Martinez-Cayuelas et al., 2022; Martinez-Cayuelas, Moreno-Vinués, Martinez-Cayuelas et al., 2024a, b; Martínez-Cayuelas et al., 2022; Mughal et al., 2020; Tatsumi et al., 2015; Tse et al., 2020), lower sleep efficiency (Benson et al., 2023; Chua et al., 2022; Fletcher et al., 2017; Martinez-Cayuelas et al., 2022; Martinez-Cayuelas, Moreno-Vinués, Martinez-Cayuelas et al., 2024a, b; Martínez-Cayuelas et al., 2022; Mughal et al., 2020; Tse et al., 2020), and longer sleep latency (Chua et al., 2022; Martinez-Cayuelas et al., 2022; Martinez-Cayuelas, Moreno-Vinués, Martinez-Cayuelas et al., 2024a, b; Martínez-Cayuelas et al., 2022; Tse et al., 2020) than their peers. More inconsistently reported impairments on sleep parameters for ASD included later bedtime (Martinez-Cayuelas et al., 2022), later sleep onset time (Martinez-Cayuelas et al., 2022), longer mean sleep bout (Mughal et al., 2020), longer mean nighttime wakings (Mughal et al., 2020), lower fragmentation index (Martinez-Cayuelas et al., 2022; Mughal et al., 2020), longer snooze time (Tatsumi et al., 2015), or longer wake after sleep onset (Tatsumi et al., 2015).

**Table 2:** Studies using wearables to assess sleep in children and adolescents – autism spectrum disorder (ASD)

Study reference	Sample characteristics	Study aim	Outcome measures	Main findings
Abel et al. (2018)	42 ASD	To examine the associations between sleep and challenging behaviours with average and day-to-day fluctuations in sleep	motion, total sleep time; wake after sleep onset	Total sleep time was negatively associated with challenging behaviours and repetitive behaviours during treatment.
Alder et al. (2020)	9 ASD, 6 TD	Exploring novel actigraphy endpoints that characterize movement events during sleep	wake and sleep time; nighttime physical movement	A strong association was found between actigraphy events and the presence and intensity of nighttime movement activity, and between actigraphy events and night wakings
Bangerter et al. (2020)	144 ASD, 41 TD	Examining the relationship between sleep and caregiver-reported behaviours in children with ASD, including the use of machine learning to identify sleep variables important in predicting anxiety in ASD.	sleep start; sleep duration; number of awakenings; sleep efficiency	Caregiver-report of sleep problems was not related to mean actigraphy measures, however, there was a relationship with variability in sleep efficiency. In addition, a relationship was found between actigraphy and some caregiver-reported measures such as, hyperactivity and anxiety
Benson et al. (2023)	21 ASD, 45 TD	Providing a comparison of behavioural, cognitive, affect-related (anxiety), and sleep profiles between the three groups	total sleep time; wake after sleep onset; sleep onset latency; sleep efficiency	Children with ASD had less sleep per night and lower sleep efficiency than their peers.
Chua et al. (2022)	40 ASD, 37 TD	Investigating sleep and its associated impact on attention in this population, using actigraphy and a computerised Continuous Performance Task	sleep efficiency; sleep latency; night-waking duration; sleep duration	Longer sleep latency and lower sleep efficiency were present among children with ASD.
Fletcher et al. (2017)	21 ASD, 29 TD	Comparing the trajectory of parent-report and actigraphy-derived sleep profiles over a period of 12–15 months.	sleep onset time; sleep offset time; sleep onset latency; sleep efficiency; sleep period duration; stability if sleep; wake after sleep onset	Children with ASD are characterised by a lower sleep efficiency.
Iwamoto et al. (2023)	26 ASD	Exploring the directionality of relationships between child sleep duration, child behaviour problems, and parenting stress in a diverse group of families of children with ASD at the daily, within-person level.	sleep duration; waketime; wake after sleep onset; number of awakenings; length of awakenings; sleep efficiency	Shorter sleep duration, on average, was associated with higher child behaviour problems
Kosaka et al. (2021)	20 ASD, 20 TD	To validate the relationship between sensory characteristics and sleep dynamics among children with ASD using Sensory Profile and actigraph	time in bed; total sleep time; sleep latency; wake after sleep onset; sleep efficiency	No differences between ASD and TD group.
Martínez-Cayuelas et al. (2022)	52 ASD, 27 TD	To compare circadian rhythms and sleep patterns in children and adolescents with ASD and a control group made up of TD children, using an ACM device.	total sleep time; sleep onset latency; wake time after sleep onset; time in bed; number of awakening after sleep onset; sleep efficiency; interdaily stability; triaxial acceleration; time in movement; variability in body position; total visible light; infrared light; blue light; thermometry, actimetry and body position; intradaily variability; midpoint of sleep; circadian function index; wrist temperature	Children with ASD had lower total sleep time, longer sleep onset latency, lower sleep efficiency; and less overall and blue light 2 h before sleep.
Martinez-Cayuelas et al. (2022)	37 ASD, 24 TD	Define sleep and circadian patterns in children and adolescents with ASD using a combination of actigraphy recordings and measurements of saliva, comparing these measures against a sex-, body mass index-, and pubertal stage-matched control group.	sleep; motor activity; time in movement; thermometry, actimetry and body position; wrist temperature	Children with ASD had later bedtime, later sleep onset time, lower total sleep time, lower sleep onset latency, lower sleep efficiency, less total and blue light two hours before sleep, less exposure to outdoor light, less total and blue light two hours after waking, and less time in movement two hours after waking.
Martinez-Cayuelas, Gavela-Pérez, Martinez-Cayuelas et al. (2024a, b)	45 ASD, 24 TD	To explore relationship between behavioural difficulties, disturbances in circadian rhythm and sleep.	sleep; motor activity; time in movement; wrist temperature	In children with ASD, but not typically developing children, somatic complaints correlated positively with both awakenings per hour and wake after sleep onset, symptoms of being withdrawn correlated positively with sleep onset latency and negatively with sleep efficiency, and symptoms of anxious/depressed correlated positively with sleep onset latency.
Martinez-Cayuelas, Moreno-Vinués, Martinez-Cayuelas et al. (2024a, b)	87 ASD, 30 TD	Comparing circadian rhythms and sleep patterns using an ACM device in ASD children, ASD children with co-occurring ADHD, and a comparison group of TD children	total sleep time; sleep onset latency; wake after sleep onset; bedtime; morning wakeup time; time in bed; number of awakenings after sleep onset; sleep efficiency	Children with ASD showed shorter total sleep time, longer sleep onset latency, lower sleep efficiency, less light (overall and blue) 2 h before and after sleep, and lower exposure to outdoor light than typical controls.
Mughal et al. (2020)	21 ASD, 45 TD	Examine the association between sleep and cognitive outcomes in children with ASD in comparison to a TD control group	bedtime; wake time; assumed sleep time; actual sleep time; sleep efficiency; sleep latency; number of mean sleep bouts; mean night waking duration; mean activity epochs; fragmentation index	Children with ASD had lower actual sleep time, lower sleep efficiency, longer mean sleep bout, longer mean nighttime wakings, and a lower fragmentation index.
Phung et al. (2019)	20 ASD	Examining actigraph-based reports of sleep quality in relation to: psychological well-being, adaptive and problem behaviours, and quality of relationships with mothers and siblings	total sleep time; sleep efficiency; number of wake episodes	More time asleep and poorer sleep efficiency were associated with more depressive symptoms and discordant sibling relationships.
Richdale et al. (2014)	27 ASD, 27 TD	Investigating relationships between sleep disturbance, psychopathology symptoms, and daytime functioning in adolescents with ASD compared to TD adolescents.	sleep onset time; wake time; sleep length; sleep latency	In children with ASD, sleep onset time was positively correlated to level of depressive symptoms.
Surtees et al. (2019)	16 ASD, 16 TD	Investigate similarities and differences in sleep difficulties, use multiple methods, between children with ASD and TD	bedtime; get-up time; sleep onset latency; wake after sleep onset; time in bed; total sleep time; sleep efficiency	No evidence that children with ASD slept for shorter periods or experienced greater durations of waking or longer sleep latencies than TD
Tatsumi et al. (2015)	31 ASD, 16 TD	Investigate the association between daytime physical activity and sleep in pre-schoolers with or without ASD	Sleep onset, sleep-end time, total sleep duration, snooze time, sleep percentage and physical activity	Sleep percentage was lower in children with ASD and snooze time was longer.
Tse et al. (2020)	78 ASD, 78 TD	Investigate whether sleep patterns differed between children with ASD and those with typical development	sleep efficiency, sleep-onset latency, length of time to fall asleep, sleep duration, and wake after sleep onset	Children with ASD had lower sleep efficiency, longer sleep onset latency, longer wake after sleep onset and shorter sleep duration than children with TD, regardless of whether it was a weekday or the weekend

ASD = autism spectrum disorder; TD = typically developing

Regarding dimensional analyses, there were negative correlations between sleep parameters on the one hand, such as total sleep time and sleep efficiency, and functioning at emotional or behavioural level on the other hand, such as challenging or hyperactive behaviours (Abel et al., 2018; Iwamoto et al., 2023), symptoms of depression or anxiety (Abel et al., 2018; Bangerter et al., 2020; Martinez-Cayuelas et al., 2024a; Phung et al., 2019; Richdale et al., 2014), and discordant sibling relationships (Phung et al., 2019). Finally, there were two studies that reported no significant associations between sleep parameters and psychiatric symptoms (Kosaka et al., 2021; Surtees et al., 2019).

The nine studies on internalizing disorders that are presented in Table 3, included children with a wide array of disorders; three studies focused on generalized anxiety disorder (GAD), three on bipolar disorder (BP), of which one also included a group of children with borderline personality disorder (BPD), one on major depressive disorder (MDD), one on obsessive-compulsive disorder (OCD), and one on posttraumatic stress disorder (PTSD). Sleep deviations as assessed with wearables were less evident in these groups than in ADHD in ASD, but still prevalent. Especially longer total sleep time seemed to be present in children with BP (Faedda et al., 2016; Huỳnh et al., 2016), BPD (Huỳnh et al., 2016), MDD (Strumberger et al., 2024), and GAD (Mullin et al., 2018), as well as longer sleep onset latency for BD (Huỳnh et al., 2016), OCD (Jaspers-Fayer et al., 2018), MDD (Strumberger et al., 2024) and GAD (Mullin et al., 2018). One study on GAD reported a positive association between sleep onset latency and symptoms of anxiety and depression as well (Mullin et al., 2018), while two other studies reported no differences for any sleep parameters in GAD (Alfano et al., 2015; Palmer et al., 2018). Moreover, nocturnal activity or nighttime awakenings seemed to be more prevalent in BP (Faedda et al., 2016) and OCD (Jaspers-Fayer et al., 2018), while PTSD was associated with a higher fragmentation (Rolling et al., 2023).

**Table 3:** Studies using wearables to assess sleep in children and adolescents – internalizing disorders

Study reference	Sample characteristics	Study aim	Outcome Measures	Main findings
Alfano et al. (2015)	39 GAD, 36 TD	Obtain a better understanding of whether and how the sleep patterns and problems of school-aged children with GAD differ from those of healthy children and to understand the extent to which cross-method correspondence exists for sleep patterns and problems within these groups	mean bedtime; bedtime consistency; total time in bed; total sleep duration; sleep onset latency; number of nighttime awakenings; wake-up time.	No group differences on any of the sleep variables.
Faedda et al. (2016)	48 BD, 42 TD	Ascertain whether objective measures of sleep and circadian rhythms can potentially aid in the differential diagnosis of paediatric BD	5 least active hours; mean nocturnal activity; percentage nocturnal activity; total sleep time; sleep onset latency; sleep efficiency	More 5 least active hours, more and a higher percentage of nocturnal activity, less total sleep time, and lower sleep efficiency in children with BD, then their typical peers.
Huỳnh et al. (2016)	18 BPD, 6 BD, 20 TD	Explore the sleep-wake cycle of euthymic adolescents with BPD or BD	average activity counts; time in bed; time awake in bed; sleep onset latency; total sleep time; sleep efficiency; final awakening; sleep time; awake time	Children with BPD or BD had longer total sleep time than typical developing children.
Jaspers-Fayer et al. (2018)	30 OCD, 30 TD	Examine subjective and objective sleep outcomes in paediatric OCD	bedtime; wake time; sleep latency; number of nighttime awakening; total sleep time; sleep efficiency	Children with OCD showed more frequent nighttime awakenings, longer sleep onset latency, than those without OCD.
Mullin et al. (2018)	26 GAD, 17 TD	Assess subjective and objective sleep parameters, and their relationships with anxiety symptomatology, among adolescents with generalized anxiety disorder and controls without any psychopathology.	total sleep time; wake after sleep onset; sleep efficiency; sleep onset latency; weekday bedtime; morning wake time; weekend bedtime; weekend morning wake time;	Children with an anxiety disorder had a longer total sleep time and longer sleep onset latency than children without an anxiety disorder. There was a negative association between sleep efficiency and level of depressive symptoms, and positive associations between sleep onset latency and levels of anxiety and of depressive symptoms.
Murphy et al. (2014)	16 BD, 4 TD	Examine if children with PBD have difficulty dissipating heat at bedtime, and if this thermoregulatory symptom is associated with longer latencies to sleep	Sleep onset latency; total sleep time; sleep efficiency	Longer sleep onset latency in paediatric BD.
Palmer et al. (2018)	75 GAD, 38 TD	To investigate the occurrence of co-sleeping and its correlates in a school-aged sample of clinically anxious and non-anxious children	sleep duration; number of awakening; wake after sleep onset; sleep onset time; sleep onset time; variability in sleep duration; variability in sleep timing	Children with an anxiety disorder did not differ on sleep characteristics from children without an anxiety disorder.
Rolling et al. (2023)	11 PTSD, 11 controls with sleep disorder	To objectively evaluate sleep and circadian rhythms in children with PTSD compared to a control population	total sleep time; sleep onset latency; wake after sleep onset; sleep efficiency; the relative amplitude; intraday variability; interday stability	Children with PTSD had higher sleep fragmentation compared to controls, with a change in sleep microarchitecture. Sleep fragmentation parameters correlated with PTSD symptomatology, insomnia, and post-traumatic nightmare severity
Strumberger et al. (2024)	29 MDD, 29 TD	Investigate the relationship between both self-reported and objective sleep variables and low-grade inflammation in children and adolescents with major depressive disorder (MDD) of moderate to severe symptom severity	Number of awakenings, wakefulness after sleep onset, total sleep time, sleep efficiency, sleep onset latency	Lower average total sleep time and higher sleep onset latency in the MDD compared to TD

BD = bipolar disorder; BPD = borderline personality disorder; GAD = generalized anxiety disorder; MDD = major depressive disorder; OCD = obsessive compulsive disorder; PTSD = posttraumatic stress disorder; TD = typically developing

Finally, six studies were identified that focused on the relation between sleep and externalizing behaviour in children (see Table 4). The main goal of these studies was to assess if wearables can be used to evaluate sleep impairments in children with externalizing behaviour, but also to assess if there were specific associations between sleep parameters and aggression (Bélanger et al., 2018) or delinquency (Stone et al., 2015). All studies indicated sleep deviations or positive associations between sleep deviations and externalizing behaviours. Firstly, total sleep time emerged as an evident marker, being negatively related to externalizing behaviours (Aronen et al., 2014; Bélanger et al., 2018; Van Dyk et al., 2016), impulsivity (Wong et al., 2018) and social problems (Bélanger et al., 2018). Additionally, shorter sleep time, as well as lower sleep efficiency, in toddlers were associated with more aggressive behaviour a year later (Bélanger et al., 2018). Duration of different sleep stages in children with disruptive behavioural disorders predicted next-day level of disruptive behaviours (Romanowicz et al., 2023), and daytime sleep duration was positively associated with delinquency (Stone et al., 2015). For children with oppositional defiant disorder (ODD) and/or conduct disorder (CD), no sleep impairments were found (Aronen et al., 2014). However, this study showed that a subgroup with ODD/CD and comorbid ADHD symptoms did show shorter time in bed, shorter total sleep time, and lower sleep efficiency, suggesting that these differences are driven by ADHD rather than by ODD/CD (Aronen et al., 2014).

**Table 4:** Studies using wearables to assess sleep in children and adolescents – externalizing behaviours

Study reference	Sample characteristics	Study aim	Outcome measures	Main findings
Aronen et al. (2014)	30 ODD/CD, 30 TD	Evaluating associations between the objectively measured sleep amount or sleep efficiency and the intensity of the psychiatric symptoms in patients with CD/ODD.	total sleep minutes; sleep efficiency; time in bed	No group differences for total group comparisons, but subgroup of ODD/CD with comorbid ADHD showed shorter total sleep, shorter time in bed and lower sleep efficiency. Both total sleep time and sleep efficiency were negative correlated with externalizing behaviour and social problems.
Bélanger et al. (2018)	82 TD	Examining reciprocal associations between aggressive behaviour and sleep in toddlers, using an objective sleep measure and both parents’ reports of children’s aggressive behaviour.	sleep efficiency; sleep duration; movement	Both lower sleep efficiency and shorter sleep duration at age 2 were associated with more aggressive behaviour at age 3.
Romanowicz et al. (2023)	10 children with disruptive behavioural disorders	To establish whether physiological data from smartwatches could predict disruptive behaviours in a sample of hospitalized children.	total sleep duration; duration of awake; sequence of sleep stages; duration of each sleep stages	The duration of REM sleep, light sleep, and deep sleep stages were longer in the night before children exhibited disruptive behaviour than in those who did not.
Stone et al. (2015)	49 children with externalising behavioural symptoms	Evaluating associations between actigraphic sleep patterns, subjective sleep quality, and daytime functioning (sleepiness, symptoms of depression, and delinquency and other conduct problems) in at-risk adolescents	nighttime sleep duration; sleep start time; final wake time; nighttime sleep minutes; nighttime wake minutes; week sleep schedule variability; daytime sleep duration	Actigraphic daytime sleep duration is positively associated with delinquency.
Van Dyk et al. (2016)	25 children with externalising behavioural symptoms	To examine the daily, bidirectional relationships between sleep and mental health symptoms in youth presenting to mental health treatment.	total sleep time	The model predicting externalizing problems indicated that for every hour increase in mean total sleep, the level of externalizing problems decreased.
Wong et al. (2018)	77 children of alcoholics, 38 TD	Comparing multiple measures of sleep and the relationships between sleep and behavioural problems in 2 groups of children	total sleep time; sleep efficiency; sleep onset latency; wake time after sleep onset	Total sleep time was negatively related to impulsivity.

ADHD = attention-deficit/hyperactivity disorder; CD = conduct disorder; ODD = oppositional defiant disorder; REM = rapid eye movement; TD = typically developing

Regarding feasibility, only few studies reported specifically whether wearables were tolerated well. Looking at the amount of usable data that was retrieved, 87% of the participants wore the actigraphy device long enough to retrieve usable data for at least 5 days, whereas the remaining 13% only had data for 4 days (Melegari et al., 2020). Importantly, there were no differences between children with and those without ADHD for the number of recorded days, indicating its usefulness in both groups.

### Assessment of the ANS

The assessment of the ANS has been a focal point in numerous studies using wearables. The ANS regulates involuntary physiological functions such as heart rate, blood pressure, digestion, and respiratory rate. To measure the ANS in children, researchers often used the following parameters: heart rate (HR), heart rate variability (HRV), blood volume pulse (BVP), or skin conductance (galvanic skin response or electrodermal activity). As can be seen in Table 5, a total of 19 studies were identified that examined ANS parameters. Most of these studies concentrated on children with ASD, highlighting the interest in understanding the physiological underpinnings of the behaviour of autistic children. Additionally, one study focused on OCD (Lønfeldt et al., 2023), one on traumatized youth (Schuurmans et al., 2020) and one on a transdiagnostic sample of youth (Naim et al., 2021). The Empatica E4 wristband emerged as the most frequently employed wearable device in these studies, but also wireless ECG chest bands were used.

**Table 5:** Studies using wearables to assess parameters of the autonomic nervous system in children and adolescents

Study reference	Sample characteristics	Study aim	Outcome measures	Main findings	Feasibility
Alban et al. (2023)	5 ASD	To investigate the potential of data acquired using wearables can detect challenging behaviours in children with ASD.	Acceleration; electrodermal activity; temperature; heart rate; blood volume pulse; challenging behaviour	Machine learning models showed high precision (0.88) and accuracy (0.99) in predicting challenging behaviour. Heart rate was the most significant contributing predictor for almost all participants. Heart rate variability was found to correlate with the occurrence of challenging behaviours	
Ali et al. (2023)	9 ASD	To develop a framework for recognizing the affective state of children with ASD using heart rate information.	Heart rate; emotion	The intra-subject classification accuracy of the emotions using heart rate data varied from 32 to 100% in the different participants.	
Anandhi et al. (2022)	10 ASD, 10 TD	To understand the hidden and unexpressed emotional state of children with ASD by using physiological signals obtained from wearable devices.	Heart rate; heart rate variability; emotion	There is a diminished heart rate variability and increased heart rate in children with ASD compared to TD children. An overall maximum emotion recognition accuracy of 84.8% and 75.3% was found for the TD and ASD group respectively, when using heart rate variability data.	
Bagirathan et al. (2021)	6 ASD, 6 TD	To identify two internal states (positive and negative) of children with ASD using physiological data.	Heart rate variability; electrocardiogram (ECG) data, emotion	An overall maximum emotion recognition accuracy of 84.7% and 81.1% was found for the TD and ASD group respectively, when using heart rate variability data. HRV data was found to be more effective than ECG data in capturing the valence states.	
Baker et al. (2018)	40 ASD	To test whether lower electrodermal activity would predict more externalizing problems in children with ASD	Electrodermal activity; externalizing problems	Low electrodermal activity during parent-child compliance interactions was related to higher parent-reported child externalizing problems.	Approximately three-quarters of the children easily tolerated an additional sensor. Some data were missing due to equipment malfunction and/or child removal of sensors.
Billeci et al. (2016)	5 ASD	To describe the implementation of a wearable technology system that can be used during naturalistic interactions between a child and a clinician during a treatment session for the monitoring of autonomic response.	EEG (alpha, beta, delta, theta, gamma) and ECG (heart rate and heart rate variability) signals	Visible changes were found in the EEG pattern during treatment elicited by interaction of the child with the therapist. ECG analysis confirmed cardiac activity is influenced by social engagement states and the ECG patterns turned out to be subjected to similar changes in the five subjects in response to changed psychological conditions.	The children did not show sensory-motor and/or behavioural issues in wearing the devices
Billeci et al. (2018)	20 ASD, 20 TD	To test the feasibility of using a wearable chest belt for the monitoring of ECG signal in toddlers with ASD during a joint attention eye-tracking task.	ECG data (heart rate; coefficient of variation (CV); Low Frequency (LF); High Frequency (HF))	In the ASD group, LF decreased from baseline to task. Increased SDNN and CV were found in toddlers with ASD compared to TD children. CV at baseline was positively correlated with initiation joint attention.	The system did not cause any kind of annoyance and all children successfully accomplished the experimental protocol without showing sensory-motor and/or behavioural issues in wearing the devices.
Costescu et al. (2021)	3 ASD	To assess whether wearables can accurately measure heart rate, which has been associated with emotional dysregulation in preschoolers with ASD.	Heart rate; emotional outbursts	Graphic analysis revealed that emotional outbursts were associated with elevated hear rate values for all three participants. There were also other moments in the therapy sessions that increased the heart rate levels.	
Fioriello et al. (2020)	12 ASD	To evaluate whether heart at can be used as a possible indicator of stress response in children with ASD.	ECG signals (heart rate); ASD symptoms	HR of children with ASD was correlated with ASD symptoms during one interactive activity; but not during other activities. ASD symptoms influenced HR variations.	
Goodwin et al. (2019)	20 ASD	To investigate whether preceding physiological and motion data measured by a wrist-worn biosensor can predict aggression to others by youth with ASD.	Blood volume pulse (BVP); Inter-Beat Interval (IBI); heart rate; heart rate variability; aggressive episodes	Aggression to others can be predicted 1 min before it occurs using 3 min of prior biosensor data with an average area under the curve of 0.71 for a global model and 0.84 for person-dependent models. Models that included physiological and motion activity features outperformed those with only temporal features.	All participants tolerated wearing the wearable and usable data were obtained in all cases
Greenlee et al. (2024)	60 ASD	To investigate whether metrics of electrodermal activity are correlated with the behaviour of children with ASD.	Frequency of peaks; Skin conductance level; body temperature; mood, social responsiveness, dysregulation, cooperation	A higher number of peaks was positively associated with autonomy and responsiveness, and negatively associated with negative mood and emotion dysregulation. The amplitude of peaks was associated with less adaptive behaviours.	
Imbiriba et al. (2023)	70 ASD	Investigate whether changes in peripheral physiology recorded by a wearable biosensor can be used to predict aggressive behaviour in youth with ASD.	Blood volume pulse; electrodermal activity; motion activity; aggressive behaviour	Machine learning analyses of peripheral physiology data yielded a mean 0.80 AUROC 3 min before aggressive behaviour onset.	8 individuals were excluded because they would not wear the biosensor
Krupa et al. (2016)	10 ASD, 10 TD	To examine whether physiological signals can predict emotional states in children with ASD.	Galvanic skin response; heart rate variability; emotional states	A model using data from galvanic skin response and heart rate variability yielded a reliable prediction of emotions in children with ASD with a success rate of 90%.	A few children were reluctant on wearing the device.
Kushki et al. (2014)	24 ASD	To explore whether arousal associated with anxiety can be detected based on cardiac activity data from children with ASD	ECG data (R–R intervals); self-report of arousal associated with anxiety	The proposed method can detect physiological arousal associated with anxiety with high sensitivity and specificity of 99% and 92% respectively.	
Nuske et al. (2019)	13 ASD	To examine whether heart rate predicts challenging behaviours in young children with ASD.	ECG data (heart rate and heart rate variability); challenging behaviours	An increase in heart rate was associated with the onset of challenging behaviours. Heart rate variability was not a significant predictor.	
Nuske et al. (2022)	32 ASD, 23 TD	To determine the validity of wearables in measuring psychosocial stress in children with ASD.	Heart rate and heart rate variability	Increased HR and decreased HRV were found during the stress task compared to the rest task.	The devices met the optimal threshold of 80% of participants wearing the monitors throughout the entire 2-h session. All children rated the wearables in the comfortable range.
Lønfeldt et al. (2023)	9 OCD	To test the feasibility of capturing OCD events using an unobtrusive wrist worn biosensor and machine learning models in a sample of adolescents with OCD.	Blood volume pulse; external skin temperature; electrodermal activity; heart rate; OCD events	OCD episodes were detected using physiological signals captured with a wearable biosensor. The tree-based models demonstrated the best performance and reached 70% accuracy. The most influential features in the models were related to blood volume pulse parameters.	One participant did not wear the wearable at all outside of the lab visits. The retention rate for wearing the biosensor in everyday life for up to 8 weeks was 78%. Participants registered 2,146 OCD events using the event tag button. Patients did not report any false or accidental registrations/tags.
Schuurmans et al. (2020)	15 PTSD	To evaluate the accuracy and predictive value of the Empatica E4 wristband by comparing it to the VU-AMS as reference golden standard while worn on both wrists in a clinical population of adolescents in residential care	High frequency (HF); Heart rate (HR); Inter beat interval (IBI); RR intervals; Low frequency (LF); Ratio between low and high frequency (LF/HF); Root mean squared differences of successive difference of intervals (RMSSD); Standard deviation of the normal-to-normal interval (SDNN);	Results of this study indicate the potential of the Empatica E4 as a practical and valid tool for research on HR and HRV under non-movement conditions	
Naim et al. (2021)	16 ADHD, 26 internalizing, 9 TD	Exploring the link between irritability and cardiovascular arousal during an in-lab standardized stop‐signal task in a trans-diagnostic sample of youth.	Blood volume pulse (BVP); heart rate (HR); heart rate variability (HRV)	This study demonstrates higher HR and lower HRV as a function of irritability during a standard cognitive inhibition task in a transdiagnostic sample of youth.	

ADHD = attention-deficit/hyperactivity disorder; ASD = autism spectrum disorder; CD = conduct disorder; OCD = obsessive compulsive disorder; ODD = oppositional defiant disorder; PTSD = posttraumatic stress disorder; TD = typically developing

The studies on cardiac activity in children with ASD mostly aimed to assess whether challenging behaviour or emotional outbursts can be predicted by parameters such as HR or HRV. Some studies included machine learning models to optimally predict the behavioural aspects. Results in several studies showed that HR was significantly associated with challenging behaviour in children with ASD (Alban et al., 2023; Costescu et al., 2021; Nuske et al., 2019). More specifically, aggression in children with ASD was found to be predicted 1 min before it occurs using 3 min of prior biosensor data (Goodwin et al., 2019; Imbiriba et al., 2023). Cardiac activity also was shown to predict affective state in children with ÀSD (Ali et al., 2023; Anandhi et al., 2022; Bagirathan et al., 2021; Krupa et al., 2016), and to be associated with anxiety (Kushki et al., 2014) and stress during social interactions (Billeci et al., 2016; Fioriello et al., 2020; Nuske et al., 2022). Finally, it was found that children with ASD showed diminished HRV and increased HR (Anandhi et al., 2022), and increased SDNN and CV (Billeci et al., 2018) compared to TD children.

Two studies on children with ASD focused on skin conductance. Main findings in these studies were that low electrodermal activity of the child during parent-child compliance interactions was related to higher parent-reported child externalizing problems (Baker et al., 2018), and that metrics of electrodermal activity were correlated with social behaviour (autonomy and responsiveness) and emotion regulation in children with ASD (Greenlee et al., 2024). In other studies, skin conductance parameters were included in the overall prediction of challenging behaviour (Alban et al., 2023; Imbiriba et al., 2023; Krupa et al., 2016).

The other two studies showed that OCD episodes can be accurately detected using physiological signals (e.g., electrodermal activity, BVP, skin temperature) captured with a wearable biosensor (Lønfeldt et al., 2023), and that a higher HR and lower HRV were found in a transdiagnostic sample of youth, as a function of irritability during a cognitive inhibition task (Naim et al., 2021).

Regarding feasibility, most studies did not report on how well the wearables were tolerated by the participants or reported no signals of sensory-motor or behavioural problems in participants wearing the devices (Billeci et al., 2016, 2018; Goodwin et al., 2019). Overall, using the wearables seemed feasible in most studies, for instance with an 80% wear rate during sessions in one study (Nuske et al., 2022). However, in some studies it was found that participants refused to wear the biosensor (Imbiriba et al., 2023; Krupa et al., 2016) or that participants removed sensors (Baker et al., 2018).

### Assessment of Motion and Activity

As presented in Table 6, a total of eight studies were identified that investigated a diverse array of motion parameters in various paediatric conditions(Cantin-Garside et al., 2020; Chu et al., 2020; Dekkers et al., 2021; Hartanto et al., 2016; Hudec et al., 2015; McGinnis et al., 2018; Merikanto et al., 2017; Patros et al., 2017). Notably, five studies focused on ADHD, one on a sample of children with an internalizing disorder, one on adolescent boys with a depressive disorder, and one on children with ASD. In all eight studies, accelerometer devices were used, varying from actigraphy to waist-worn belts.

**Table 6:** Studies using wearables for assessment of motion and activity parameters

Study reference	Sample characteristics	Study aims	Outcome measures	Main findings	Feasibility
Chu et al. (2020)	49 ADHD, 14 TD	To determine potential indicators extracted from an actigraphy device to validate them for diagnosis of ADHD	Actigraphy activity; computer-operated continuous performance test (CPT)	ADHD participants showed higher levels of activity (actigraphy) than TD participants.	Activity amounts (actigraphy) were positively related to impulsive behaviour (CPT)
Dekkers et al. (2021)	36 ADHD, 24 TD	To examine whether activity level changes in children with ADHD and TD children occur as a function of differences in cognitive processing demands	Intensity of movement; activity level; gross motor activity; memory; working memory	Children with ADHD were motorically more active under all working memory conditions relative to TD children. The increase in activity as a consequence of cognitive demand was similar for all experimental conditions	
Hartanto et al. (2016)	26 ADHD, 18 TD	To examine the relationship between motor activity and accuracy in cognitive control performance in ADHD and TD children.	Intensity of movement; frequency of movement; cognitive control	The ADHD group demonstrated more intense activity than the TD group during correct (but not error) trials. More intense movement was associated with better performance in the ADHD, but not TD group.	
Hudec et al. (2015)	19 ADHD, 18 TD	To examine the relationship between working memory and motor activity	Frequency, intensity and duration of finite motor movements; working memory	Results indicated that children in the ADHD group exhibited greater activity compared to children in the TD group. Further, both groups exhibited the greatest activity during conditions with high working memory demands.	
Patros et al. (2017)	15 ADHD, 17 TD	To examine activity changes across control and executive functioning tasks that differ with regard to demands placed on working memory and self-control processes.	Frequency, intensity, and duration of motor activity; working memory	Results indicated that boys with ADHD, relative to TD boys, exhibited greater motor activity across tasks, and both groups’ activity was greater during working memory tasks relative to control tasks.	
McGinnis et al. (2018)	21 internalizing, 40 TD	To examine the validity of a wearable sensor for identifying children with anxiety disorders during a mood induction task	Motion variables; externalizing and internalizing symptoms;	Five of six movement variables were significantly greater for children with internalizing diagnoses than TD children. All movement variables were correlated with externalizing symptoms, but not with internalizing symptoms.	
Merikanto et al. (2017)	8 depression, 9 TD	To study whether circadian indicators based on actigraphic data differ between depressed and healthy adolescent boys.	Motion variables	Reduced activity amplitudes and shorter periods of rest-activity cycles were found among depressed adolescent boys as compared to healthy controls	
Cantin-Garside et al. (2020)	11 ASD	To evaluate the performance of different machine learning algorithms to detect self-injurious behaviour in children with ASD using wearables	Movement data; self-injurious behaviour	The results support the feasibility of detecting a range of self-injurious behaviours in individuals with ASD using wearable sensors (accelerometers) with high accuracy (97–99%) and specificity (> 0.80).	Two participants did not want to wear all body sensors

ADHD = attention-deficit/hyperactivity disorder; ASD = autism spectrum disorder; TD = typically developing

In all five studies that included participants with ADHD (Chu et al., 2020; Dekkers et al., 2021; Hartanto et al., 2016; Hudec et al., 2015; Patros et al., 2017), the authors aimed to examine the relationship between motor activity on the one hand and cognitive functions such as working memory, cognitive control, and executive functioning on the other hand. The findings from these studies collectively indicate that children with ADHD exhibit greater motor activity compared to typically developing (TD) children across various tasks, suggesting higher levels of hyperactivity. However, none of these studies aimed to examine the validity of using actigraphy to assess hyperactivity by comparing it to other instruments. Further, the studies highlighted that higher motor activity was associated with increased cognitive demand. For instance, a study found that more intense movement was correlated with better performance in the ADHD group (Hartanto et al., 2016). One of the explanations for the latter is the suggestion that children with ADHD use movement to self-regulate alertness. Contrarily, one study showed that the amount of activity was positively related to impulsive behaviour on a computerized task (Chu et al., 2020).

The other three studies either aimed to detect self-injurious behaviours in individuals with ASD using accelerometers (Cantin-Garside et al., 2020), to detect altered motion patterns as indications of anxiety during a mood induction task (McGinnis et al., 2018), or to assess the rest-activity rhythm in depressed adolescent boys (Merikanto et al., 2017). One of the main findings within the study on children with ASD, was that an analysis of data obtained by wearable sensors could reveal self-injurious behaviour with high accuracy and specificity by using machine learning methods. Regarding feasibility, it should be noted that a minority (2 out of 11) of the participants did not want to wear all body sensors (Cantin-Garside et al., 2020). Furthermore, the study on children with internalizing problems, showed that motion data were associated with parent-reported child symptoms of externalizing behaviour and clinician-reported child internalizing diagnosis (McGinnis et al., 2018). Lastly, it was found that depressed adolescents boys showed lower activity levels and reduced amplitudes as compared to healthy controls when analysing actigraphy data (Merikanto et al., 2017).

### Assessment of Brain Activity and Eye Gazing

The investigation of other physiological parameters using wearable technology has provided a total of five studies, summarized in Table 7. Three studies on brain functioning were performed in participants with ADHD, and one in participants with ASD. The assessment of brain activity was performed using mobile electroencephalography (EEG) and functional near-infrared spectroscopy (fNIRS) devices. Lastly, the fifth study focused on social attention in children with ASD, using a wearable head-mounted eye-tracker.

**Table 7:** Studies using wearables to assess brain activity and eye gazing in children and adolescents

Study reference	Sample characteristics	Study aims	Outcome measures	Main findings	Feasibility
Chu et al. (2020)	49 ADHD, 14 TD	To determine potential indicators extracted from a mobile electroencephalography (EEG) device to validate them for diagnosis of ADHD	EEG values (attention and meditation); computer-operated continuous performance test (CPT)	Most correlations between EEG and CPT were not significant, except for a significant correlation of attention with parameters of interstimulus interval change.	
McCabe et al. (2023)	112 ADHD, 255 TD	To replicate previous ADHD subtype findings from multichannel EEG studies using a frontal, single-channel, dry-sensor EEG device	EEG values (delta, theta, alpha and beta power)	There were increases in frontal delta and theta power in children with ADHD (combined presentation) compared to children with ADHD (inattentive presentation) and TD children. Results regarding alpha power were inconsistent, whereas beta power did not differ between groups.	
Ortuño-Miró et al. (2023)	15 ADHD, 15 TD	To develop a functional near-infrared spectroscopy (fNIRS)-based methodological approach for effective identification of ADHD boys	Shallow-Signals; Clean (or corrected) signal	Very-low frequency fNIRS fluctuations induced/modulated by a rhythmic mental task accurately differentiate ADHD boys from non-ADHD controls.	One boy with ADHD and two from the TD control group were excluded due to NIRS signal quality issues.
Barreto et al. (2024)	12 ASD, 16 TD	To investigate the prefrontal cortex (PFC) activity using fNIRS of young children with ASD while they watched social and non-social video clips	Cerebral hemodynamics; ASD symptoms	Children with ASD exhibit distinct activation patterns for social and non-social stimuli. PFC activity of ASD children was significantly higher for social stimuli at medial PFC. Moreover, this activity was also consistently correlated with ASD symptoms, and higher activation of the same brain area only during social video viewing was associated with more ASD symptoms. Lastly, PFC activity was higher in the ASD group than the TD group in the social condition.	
Clin et al. (2024)	18 ASD, 36 TD	To explore the potential influence of partner familiarity or of the conversational topic in children with ASD on social attention	Eye gaze behaviour; skin conductance	Children with ASD did not differ from TD children in their overall social attention on their interactional partner’s eyes, nor in their overall electrodermal activity.	

ADHD = attention-deficit/hyperactivity disorder; ASD = autism spectrum disorder; TD = typically developing

Two studies observed that participants with ADHD exhibited distinct neural patterns compared to non-ADHD controls (Ortuño-Miró et al., 2023). The fNIRS study effectively differentiated ADHD boys from non-ADHD controls (Ortuño-Miró et al., 2023), and there were increases in frontal delta and theta power in children with ADHD (combined presentation) compared to TD children (McCabe et al., 2023). The study on social attention found no differences between young children with ASD and their TD peers on measures of eye tracking or skin conductance (Clin et al., 2024).

Regarding the tolerability of using a wearable fNIRS system in children, one study reported three dropouts (10%) due to NIRS signal quality issues (Ortuño-Miró et al., 2023).

### Studies Relevant to Forensic Youth Care and Rehabilitation

Of the 85 studies included in this review, 10 studies (11.8%) focused on youth (at risk of) offender populations or symptoms directly relevant to forensic mental health care. These studies addressed externalizing behaviours, CD, ODD, impulsivity, delinquency, aggression, or were conducted in residential or forensic psychiatric settings. Together, they highlight the potential utility of wearable technologies in detecting early warning signals of behavioural escalation and supporting tailored interventions in high-risk youth.

Several studies examined the relationship between objectively measured sleep parameters and externalizing behaviours. Aronen et al. (2014) assessed sleep in youth with CD or ODD and found that those with comorbid ADHD had shorter total sleep time and lower sleep efficiency. These sleep impairments were negatively correlated with externalizing symptoms and social difficulties. Bélanger et al. (2018) reported that lower sleep efficiency and shorter sleep duration at age two predicted more aggressive behaviour one year later, based on actigraphy data in a community sample. Romanowicz et al. (2023), working in a forensic psychiatric setting, found that children who exhibited disruptive behaviour had experienced longer durations of REM, light, and deep sleep the preceding night, suggesting the potential predictive value of sleep staging data from wearable smartwatches.

Other studies found associations between sleep and behavioural symptoms in at-risk populations. Stone et al. (2015) showed that increased daytime sleep duration, as measured by actigraphy, was linked to greater levels of delinquency in adolescents with externalizing symptoms. Similarly, Van Dyk et al. (2016) demonstrated a bidirectional relationship between total sleep time and externalizing problems in a mental health population, with greater sleep predicting reduced symptoms the following day. Wong et al. (2018) found that shorter total sleep time was associated with higher levels of impulsivity in children of alcoholics, a group at elevated risk for behavioural dysregulation.

In addition to sleep-focused research, several studies explored the use of wearable devices to assess autonomic nervous system functioning in forensic-relevant contexts. Schuurmans et al. (2020) validated the use of the Empatica E4 wristband in adolescents with PTSD living in a secure residential facility, finding that the device produced HRV data comparable to standard ECG measures. Rolling et al. (2023) also studied youth with PTSD and found that greater sleep fragmentation, measured via actigraphy, was associated with PTSD symptom severity and post-traumatic nightmares.

Two studies on youth with autism spectrum disorder (ASD) further demonstrated the predictive value of wearable-derived physiological data for managing aggression. Goodwin et al. (2019) showed that aggressive episodes could be predicted up to one minute in advance based on biosensor data, including heart rate and movement patterns. Imbiriba et al. (2023) similarly used wearable data and machine learning to accurately predict aggressive behaviour in youth with ASD in an inpatient setting.

Collectively, these studies support the clinical potential of wearables in the context of forensic youth care. Wearables may help identify risk states, such as rising arousal or deteriorating sleep, that precede behavioural incidents. In institutional settings where self-report may be limited, physiological data from wearable devices can inform early intervention, risk assessment, and the development of personalized care strategies aimed at reducing behavioural crises and supporting rehabilitation.

### Intervention Studies

The search strategy within this scoping review yielded no studies that used wearables as part of an intervention for children or adolescents for mental health purposes. However, it was noted during the first phase of the screening that many intervention studies used wearables to monitor the effects of an intervention. For instance, a study using a randomized crossover trial examined the efficacy of a novel mattress-based technology in improving sleep in children with ASD. Within this study each arm included two weeks of sleep data collection using actigraphy to analyse whether there were greater improvements in sleep duration and sleep efficiency in the experimental condition (Frazier et al., 2017). Although including studies that used wearables to measure the effects of interventions in youth falls outside this review’s scope, this observation provides insight into future directions for using wearables in research and clinical practice.

## Discussion

This scoping review highlights a growing body of research utilizing wearable technologies to monitor physiological markers relevant to child and adolescent mental health. The included studies demonstrate that wearables are commonly used for the assessment of sleep, motion, and ANS parameters, particularly in youth with ADHD, ASD, and internalizing problems.

The first aim of this scoping review was to provide a comprehensive overview of how wearable technologies are currently used to assess physiological parameters in children and adolescents with mental health problems. A total of 85 studies met the inclusion criteria, reflecting a growing body of research in this area. Notably, over one-third of these studies were published in 2022 or later, underscoring the rapid expansion of interest in wearable-based monitoring in paediatric mental health care. The included studies primarily focused on the use of wearables to assess sleep, heart rate variability, and physical activity across a range of clinical populations, including youth with ADHD, ASD, internalizing disorders, and externalizing behaviours. Most commonly, actigraphy devices were used to monitor sleep, while wrist-worn biosensors and ECG chest straps were employed to capture autonomic nervous system activity. These technologies enabled the collection of objective, real-time physiological data in naturalistic settings, offering valuable insights into dysregulation that may not be observable through traditional assessment methods. The breadth and recent growth of this literature highlight the increasing relevance of wearable technology as a tool for enhancing diagnostic precision and clinical decision-making in youth mental health services.

The second aim was to summarize how physiological data from wearables relate to mental health symptoms in youth. The review shows that wearables are useful tools to measure such physiological correlates of psychopathology in youth. Physiological data collected by wearables were linked to various psychological symptoms, including inattention, hyperactivity, sleep disturbances, anxiety, and aggression. For example, disrupted sleep patterns were more frequently observed in children with ADHD and ASD, and heart rate variability was found to be related to both internalizing and externalizing symptoms. These findings reinforce the potential of wearable devices to enhance the ecological validity of mental health assessment, particularly when used alongside other methods such as self-report or clinical interviews. Wearables may offer an especially valuable addition in youth populations, where challenges in emotional awareness or verbal reporting often hinder traditional assessment approaches.

Thirdly, we examined the feasibility of using wearables in youth to accurately assess mental health problems. Most of the studies did not report on how well the wearables were tolerated by the participants or reported no indications of problems in participants wearing the devices. However, in some studies, several children refused to participate because they did not want to wear the wearable or refused to wear all sensors (Baker et al., 2018; Imbiriba et al., 2023; Krupa et al., 2016). Overall, participants seemed to be willing to use the wearables, even in children with ASD who often are hampered by an increased sensitivity to wearing external stimuli on their skin. Interestingly, the validity of wearable technology compared to established physiological measures, such as the VU AMS (De Geus et al., 1995), is rarely assessed. The field appears to have shifted away from validation studies toward direct implementation.

In addition to their clinical applications, this review highlights the emerging role of wearables in the context of juvenile justice, where they may play a crucial role in both prevention and intervention. The juvenile justice system faces significant challenges in managing youth with mental health and behavioural issues, often lacking objective, real-time data to guide interventions. Wearable devices offer a unique opportunity for continuous monitoring in forensic settings. By detecting early signs of distress, such as increased arousal or disrupted sleep, wearables could help identify youth at risk of engaging in externalizing behaviours like aggression or substance use. This data could inform tailored interventions, offering timely, targeted support to reduce the likelihood of relapse or further involvement in the justice system.

While the overall number of studies in the populations of youth at risk of externalizing behaviour remains limited (10 out of 85), several promising directions emerged. These studies focused on youth with conduct problems, disruptive behaviour disorders, or those receiving treatment in residential or secure psychiatric settings. For example, Schuurmans et al. (2020) validated the Empatica E4 wristband in adolescents with PTSD in a secure residential facility, while Imbiriba et al. (2023) used wearable biosensors to predict aggression in inpatient youth with ASD. These findings suggest that wearables can be feasibly implemented in high-risk environments and may provide clinically relevant data for early detection and intervention.

Importantly, youth in the juvenile justice system often present with complex psychosocial backgrounds, including trauma exposure, low academic achievement, family instability, and high levels of emotional and behavioural dysregulation (Vaughn et al., 2014). These characteristics are not fundamentally different from those observed in youth with externalizing behaviour problems in general populations. In fact, externalizing behaviours such as aggression, defiance, and rule-breaking, are among the most common childhood adjustment problems and are strong predictors of later mental health difficulties (Reef et al., 2010). This overlap underscores the relevance of findings from broader clinical samples for forensic contexts and supports the generalizability of wearable-based monitoring approaches across settings.

Given the complex needs of youth in forensic care, including emotion regulation difficulties and elevated stress reactivity, wearable-based monitoring may offer unique clinical value. Integration of real-time physiological data with behavioural observations could support just-in-time adaptive interventions, biofeedback training, and relapse prevention strategies. For instance, studies have shown that aggression and emotional outbursts can be predicted up to one minute in advance using biosensor data, enabling proactive de-escalation. Similarly, sleep disturbances that are frequently observed in these populations were associated with increased delinquency and impulsivity, underscoring the potential of sleep monitoring to inform risk assessment and care planning. Moreover, wearables may assist in the reintegration of institutionalized youth by promoting emotional awareness and self-regulation. Providing youth with feedback on their physiological states could foster autonomy and behavioural insight, ultimately contributing to reduced recidivism and improved long-term outcomes. As this area continues to develop, it presents an exciting opportunity to bridge the gap between technology, youth mental health, and juvenile justice.

Lastly, we were surprised to find no studies that used wearables as part of an intervention for children or adolescents in clinical settings. Given the potential of wearables to discriminate between various mental health disorders, which suggests adequate sensitivity to assess deviations and changes over time, their use (other than purely monitoring) in interventions could provide valuable insights. For example, wearables could be used to assess the effects of an intervention on alleviating symptoms, such as improving sleep, by monitoring deviations in sleep patterns. This would allow for early detection of issues without immediate intervention from clinicians, making the process not only objective and accessible but also time efficient.

The current review demonstrates several strengths, including a comprehensive search and selection process. It employed a systematic and rigorous strategy, ensuring that a wide range of relevant studies were included from multiple databases, which strengthens the robustness of the findings. Additionally, the review had clearly defined inclusion criteria, focusing on studies with methodologically sound designs (e.g., randomized controlled trials, cohort studies) and examining youth populations using wearable devices for mental health monitoring or intervention. Further, we specifically chose to focus on studies that included minors only (no adults of 18 years or older). This enables us to draw conclusions on the potential of wearables to be used in juvenile mental health care, which is often separate from the adult mental health care system. Excluding studies that consisted of a mixed sample of both adolescents and young adults, prevented us from drawing conclusions on results that were driven by an increase in maturity or altered physiological response pattern in (young) adults. Our review also emphasizes the emerging role of wearable technology in paediatric mental health care, a rapidly advancing area that enhances the timeliness and impact of the review’s findings. Lastly, the review highlights important research gaps, such as the lack of studies using wearables as interventions and the need for larger trials, offering a valuable roadmap for future exploration in this field.

Despite its comprehensive scope, due to the available studies, the generalizability of the findings and the strength of the conclusions of the current review are somewhat limited. First, although wearables show promise for youth mental health, most studies were conducted in controlled research settings, limiting their generalizability to real-world clinical or forensic environments. Second, the majority of included studies focused on assessment rather than intervention, with very few studies examining how wearable data could be used in real-time or therapeutically. Third, methodological heterogeneity such as variation in device types, outcome measures, and data processing, complicates synthesis and limits direct comparisons across studies. Additionally, only a small number of studies addressed externalizing behaviours or included youth in forensic or residential settings, despite this being a specific focus of the review. Age-related and developmental differences were often underexplored, even though wearability, compliance, and physiological responses may vary across childhood and adolescence. Moreover, few longitudinal studies were identified, limiting conclusions about the long-term utility or impact of wearable use in these populations. Finally, very limited evidence was found regarding the integration of wearable data into clinical decision-making, highlighting a key gap in translational research.

Despite some limitations, this scoping review provides a comprehensive and timely overview of how wearable technologies are currently used to assess physiological functioning in children and adolescents with mental health problems. By systematically mapping the literature, we identified promising applications of wearables for monitoring sleep, stress, and activity patterns across a wide range of clinical populations, including those in forensic and high-risk settings. These technologies offer a unique opportunity to move beyond self-report and clinician observation, enabling real-time, objective, and ecologically valid assessments of internal states that are often difficult for youth to articulate.

Importantly, the review reveals a critical gap: although wearables are increasingly used for assessment, their integration into intervention strategies remains virtually unexplored. This represents a missed opportunity, particularly in populations where early detection and timely support are essential. Future research should prioritize the development and evaluation of wearable-based interventions, including just-in-time adaptive feedback, biofeedback training, and relapse prevention tools. Such innovations could transform the way we support vulnerable youth both in clinical and forensic contexts, by promoting autonomy, emotional regulation, and long-term resilience.

In light of the acknowledged limitations, such as limited generalizability, methodological heterogeneity, and the scarcity of validation and intervention studies, the question arises: Where do we currently stand regarding the evidence base for wearable technologies in youth mental health? Although the literature demonstrates feasibility and potential clinical value, the evidence remains preliminary. The field can best be described as promising but not yet practice-ready, reflecting an exploratory phase driven by conceptual enthusiasm rather than empirical consensus. This review should therefore be viewed as a step forward towards mapping the field rather than providing definitive evidence for implementation. Moving forward, progress will depend on more rigorous validation against established physiological benchmarks, longitudinal and intervention-based designs, and greater methodological standardization to enhance comparability across studies. Equally important is implementation research that addresses feasibility, acceptability, and ethical considerations in real-world settings. Strengthening these areas will lay the foundation for the responsible and functional integration of wearable technologies into youth mental health care. As the technology continues to evolve, realizing its full potential will require interdisciplinary collaboration, ethical awareness, and a commitment to translating data into meaningful, individualized, and youth-centred support.

## Supplementary Information

Below is the link to the electronic supplementary material.

Supplementary File 1 (DOCX 14.3 KB)

Supplementary File 2 (DOCX 62.8 KB)

## Data Availability

All data and materials relevant to this scoping review are either included within the manuscript or available upon reasonable request from the corresponding author. This includes: The full search strategy for each database; Inclusion and exclusion criteria; Data extraction templates; Summary tables of included studies. No primary data were generated or analyzed during this review.
