# Supplementary material for: Using Wearables in Mental Health Care for Children and Adolescents: A Scoping Review
Source: Res Child Adolesc Psychopathol. 2026 Jan 26;54(1):16. doi: 10.1007/s10802-025-01408-9 (PMC12835090; doi:10.1007/s10802-025-01408-9)
Supplement: Supplementary file 1 — (DOCX 14.3 KB) [file 10802_2025_1408_MOESM1_ESM.docx]

## *Search strategy*

Four databases were searched (PubMed, PsycINFO, Web of Science and Embase) in August 2024. Keyword searches in these databases consisted of a combination of population search terms (youth AND mental health AND technology (wearables). Population search terms included:

baby OR babies OR infant* OR toddler* child* OR kids OR adolescen* OR teen* OR young OR youth OR juvenile OR minor OR paediatric OR paediatric.

AND

“mental health” OR behaviour* OR behaviour* OR psychologi* psychiatric OR sleep* OR stress

AND

wearable OR watch* OR wristband OR smartwatch* OR smartband OR “fitness tracker” OR “activity tracker” OR "health tracker” OR biosensor OR device* OR ambulatory OR sensing OR biofeedback OR neurofeedback OR "neuro feedback" OR mobile OR monitoring OR heart OR BPM OR electrodermal OR Arousal OR “skin temperature” OR “respiratory rate” OR “blood pressure” OR "body temperature" OR actigraph* OR actimet* OR biosignal* OR biomarker* OR signal* OR *physiological ECG OR electrocardio* OR PPG OR photoplethysmo* OR electroderm* OR EDG OR EDA “cardiac output” OR “stroke volume”. See supplementary materials for a complete overview of the search strategy.
