# Supplementary material for: Using Wearables in Mental Health Care for Children and Adolescents: A Scoping Review
Source: Res Child Adolesc Psychopathol. 2026 Jan 26;54(1):16. doi: 10.1007/s10802-025-01408-9 (PMC12835090; doi:10.1007/s10802-025-01408-9)
Supplement: Supplementary file 2 — (DOCX 62.8 KB) [file 10802_2025_1408_MOESM2_ESM.docx]

| **Table s1** *Summary of Studies on the Use of Wearables in Paediatric Mental Health Care* | | | | | | |
| --- | --- | --- | --- | --- | --- | --- |
| **Study Reference** | **Sample characteristics** | **Recruitment source** | **Percentage male** | **Age (years)** | **Domain** | **Type of wearable** |
|  |  |  |  | ***range/mean*** |  |  |
| Becker, Langberg [16] | 162 ADHD, 140 TD | Educational and Community-Based | 65% ADHD, 45% TD | 13 | Sleep | Actigraphy device (ActiGraph GT9X Link) |
| Bergwerff, Luman and Oosterlaan [17] | 63 ADHD, 61 TD | Clinical and Medical Institutions | 75% ADHD, 51% TD | 6-13 | Sleep | Actigraphy device (Actiwatch 2; Philips Respironics, Murrysville, PA, USA) |
| Bundgaard, Asmussen [18] | 24 ADHD, 25 TD | Database | 79% ADHD, 80% TD | 2-3 | Sleep | Actigraphy device (wActisleep-BT and wGTX-BT (Actigraph, Penacola, FL, USA)) |
| Cremone-Caira, Root [19] | 11 ADHD, 15 TD | Databased & Education and community-based | 82% ADHD, 67% TD | 8,27 (1,10), 8,23 (1,10) | Sleep | Actigraphy device (Actiwatch Spectrum wristwatches (Spectrum 2; Philips Respironics)) |
| Cusick, Langberg [20] | 162 ADHD, 140 TD | Educational and Community-Based | 65% ADHD, 44% TD | 12-14 | Sleep | Actigraphy device ( ActiGraph GT9X Link) |
| Faedda, Ohashi [21] | 44 ADHD, 42 TD | Clinical and Medical Institutions | 75% ADHD, 55% TD | 5-18 | Sleep (twice) | Belt-worn Actigraphy device (mini-motionlogger or motionlogger watch, Ambulatory Monitoring, Inc.) |
| Fredrick, Yeaman [22] | 140 ADHD, 162 TD | Educational and Community-Based and Clinical and Medical Institutions | 55% total | 12-14 | Sleep | Actigraphy device (ActiGraph GT9X Link) |
| Hazari, Joseph [23] | 20 ADHD, 20 TD | Clinical and Medical Institution | 90% ADHD, 90% TD | 7-12 | Sleep | Actigraphy device |
| Neto and Nunes [24] | 42 ADHD + epilepsy, 21 ADHD, 21 TD | Clinical and Medical Institutions | 64 % ADHD + epilepsy, 62% ADHD, 71% TD | 7-18 | Sleep | Actigraphy device (Micro Motionlogger, Ambulatory Monitoring, Inc., NY, USA & Act1 (EMSA Digital Actigraph, Rio de Janeiro, Brazil) |
| Kim, Kim [25] | 79 ADHD, 1011 TD | Educational and Community-Based | 70% ADHD, 51% TD | 9-11 | Sleep | Fitbit Wearable Wrist Tracker (Google LLC) |
| Knight and Dimitriou [26] | 18 ADHD, 20 TD | Educational and Community-Based | 79% ADHD, 50% TD | 5-11 | Sleep | Actigraphy device (MotionWatch 8 (CamNTech, Cambridge, UK)) |
| Langberg, Breaux [27] | 162 ADHD, 140 TD | Educational and Community-Based | 55% total | 12-14 | Sleep | Actigraphy device (Gt9x LInk) |
| Lee, Jeong [28] | 37 ADHD, 32 TD | Clinical and Medical Institutions | 100% total | 7-12 | Sleep | Actigraphy device (Actiwatch;  MiniMitter, Bend, OR, USA) |
| Miano, Amato [29] | 30 ADHD, 25 TD | Clinical and Medical Institution | 70% ADHD, 52% TD | 10,5 (2,07), 10,3 (1,54) | Sleep | Actigraphy device (Philips Respironics, Actiwatch 2) |
| Melegari, Vittori [30] | 25 ADHD, 21 TD | Clinical and Medical Institution | 84% ADHD, 64% TD | 4-6 | Sleep | Actigraphy device (Watch-Motionloggers from Ambulatory Monitoring,  Inc. (Ardsley, NY)) |
| Moreau, Rouleau and Morin [31] | 41 ADHD, 41 TD | Educational and Community-Based | 59% ADHD, 59% TD | 6-13 | Sleep | Actiwatch-64® (Mini-Mitter Co., Inc., Bend, OR) |
| Sanabra, Gómez-Hinojosa [32] | 60 ADHD, 60 TD | Clinical and Medical Institution | 57% ADHD, 57% TD | 6-16 | Sleep | ActiSleep, ActiGraph (Pensacola, FL, USA) |
| Sidol, Becker [33] | 43 ADHD | Clinical and Medical Institution | 54% ADHD | 6-13 | Sleep | Actigraphy device (The Micro‐Motionlogger SleepWatch® (Ambulatory Monitoring, Inc., Ardsley, NY)) |
| Tonetti, Occhionero [34] | 22 ADHD | Not provided | 82% ADHD | 8,8 (1,77) | Sleep | Actigraphy device, the Actiwatch AW-64 (Cambridge Neurotechnology Ltd., Cambridge, UK) |
| Waldon, Begum [35] | 24 ADHD, 24 TD | Not provided | 88% ADHD, 88% TD | 6-12 | Sleep | Actigraphy device (Micro-Mini Octagonal Basic Motionlogger Actigraph (Ambulatory Monitoring, Ardsley, NY, USA)) |
| Zerón-Rugerio, Carpio-Arias [36] | 60 ADHD, 60 TD | Clinical and Medical Institutions | 57% ADHD, 57% TD | 6-16 | Sleep | ActiSleep (ActiGraph, Pensacola, FL, USA) |
| Ziegler, Kaiser [37] | 24 ADHD, 33 TD | Clinical and Medical Institutions | 83% ADHD, 67% TD | 6-12 | Sleep | ActiGraph LLC, Pensacola, FL, USA, model: wGT3X-BT |
| Abel, Schwichtenberg [38] | 42 ASD | Clinical and Medical Institutions | 81% ASD | 2-10 | Sleep | Actigraphy device (Micromini-motionlogger, Ambulatory Monitoring, Incorporated) |
| Alder, Ye [39] | 9 ASD, 6 TD | Educational and Community-Based | 73% total | 3-10 | Sleep | Actigraphy device (Actiwatch 2 Spectrum Actiwatch (Philips Respironics)) |
| Bangerter, Chatterjee [40] | 144 ASD, 41 TD | Educational and Community-Based | 77,8% ASD, 74% TD | ≥6 | Sleep | AmbulatoryMonitoring, Inc. (AMI) Motionlogger (FDA-approved Medical Device [510(k): K854030]) |
| Benson, Mughal [41] | 21 ASD, 45 TD | Educational and Community-Based | 80% ASD, 51% TD | 6-15 | Sleep | Actigraphy device (CamNTech Motionware Actiwatch 8 (CamNTech, 2019)) |
| Chua, Neoh [42] | 40 ASD, 37 TD | Not provided | Not provided | 6-13, 6-12 | Sleep | Actigraphy device (MotionWatch8; CamNTech, Cambridge, UK) |
| Fletcher, Foster-Owens [43] | 21 ASD, 29 TD | Database | 48% ASD, 81% TD | 6;2-12;10 | Sleep | Actigraphy device (Actiwatch-2; Respironics Actiwatch 64, USA) |
| Iwamoto, Neece [44] | 26 ASD | Database | 73% ASD | 3-5 | Sleep | ActiGraph GT3XP-BTLE accelerometer |
| Kosaka, Kawatani [45] | 20 ASD, 20 TD | Clinical and Medical Institutions | 85% ASD, 60% TD | 3-6 | Sleep | Actigraphy device (Actiwatch Spectrum Plus, Philips Respironics, Inc., the USA) |
| Martínez-Cayuelas, Rodríguez-Morilla [46] | 52 ASD, 27 TD | Clinical and Medical Institution | 90% ASD, 74% TD | 6-18 | Sleep | Ambulatory Circadian Monitoring (ACM) device (Kronowise®) |
| Martinez-Cayuelas, Gavela-Pérez [47] | 37 ASD, 24 TD | Clinical and Medical Institution | 92% ASD, 75% TD | 5-18 | Sleep | Ambulatory Circadian Monitoring (ACM) device (Kronowise®) |
| Martinez-Cayuelas, Gavela-Pérez [48] | 45 ASD, 24 TD | Clinical and Medical Institution | 89% ASD, 71% TD | 5-18 | Sleep | Ambulatory Circadian Monitoring (ACM) device (Kronowise®) |
| Martinez-Cayuelas, Moreno-Vinués [49] | 87 ASD, 30 TD | Clinical and Medical Institution | 89% ASD | 6-18 | Sleep | Ambulatory Circadian Monitoring (ACM) device (Kronowise®) |
| Mughal, Hill [50] | 21 ASD, 45 TD | Educational and Community-Based | 81% ASD, 51% TD | 6-12 | Sleep | Actigraphy device (CamNTech Actiwatch 8) |
| Phung, Abdullah and Goldberg [51] | 20 ASD | Education and Community Based | 80% ASD | 11-20 | Sleep | Actigraphy device (Micro Motionlogger Sleep Watch; Ambulatory Monitoring Inc., Ardsley, NY) |
| Richdale, Baker [52] | 27 ASD, 27 TD | Education and Community Based Database | 82% ASD, 82% TD | 15,5 (1.3) | Sleep | Actigraphy device (MicroMini Motionlogger, Ambulatory Monitoring Inc. (AMI), Ardsley, NY, USA) |
| Surtees, Richards [53] | 16 ASD, 16 TD | Educational and Community-Based | 63% ASD, 63% TD | 5-14 | Sleep | Actiwatch 2 (Phillips Respironics) |
| Tatsumi, Mohri [54] | 31 ASD, 16 TD | Clinical and Medical Institution | 81% ASD, 63% TD | 4-6;9 | Sleep | Actiwatch 2 (Cambridge Neurotechnology Ltd., Cambridge, UK) |
| Tse, Yu and Lee [55] | 78 ASD, 78 TD | Educational and Community-Based | 80% ASD, 80% TD | 8-12 | Sleep | ActiGraph GT3X |
| Alfano, Patriquin and De Los Reyes [56] | 39 GAD, 36 TD | Educational and Community-Based | 48% total | 6-11 | Sleep | Actigraphy device (Micro Motionlogger Sleep Watch,Ambulatory Monitoring, Inc., Ardsley, NY, USA) |
| Faedda, Ohashi [21] | 48 BP, 42 TD | Clinical and Medical Institutions | 48% BD, 55% TD | 5-18 | Sleep | Belt-worn Actigraphy device (mini-motionlogger or motionlogger watch, Ambulatory Monitoring, Inc.) |
| Huỳnh, Guilé [57] | 18 BPD, 6 BD, 20 TD | Clinical and Medical Institutions | 17% BPD, 33% BD, 35% TD | 12-17 | Sleep | Actigraphy device (AW-64 Model, MiniMitter, Bend, OR) |
| Jaspers-Fayer, Lin [58] | 30 OCD, 30 TD | Clinical and Medical Institution | 40% OCD, 40% TD | 8-18 | Sleep | Fitbit Flex (Fitbit, Inc., San Francisco, USA) |
| Mullin, Pyle [59] | 26 GAD, 17 TD | Clinical and Medical Institution | 41% GAD, 35% TD | 12-18 | Sleep | Actiwatch-2 actigraphs (Philips Respironics, Murrysville, PA) |
| Murphy, Frei and Papolos [60] | 16 BD, 4 TD | Educational and Community-Based | Not provided | 5-12/7-9 | Sleep | Actiwatch-L and Vitalsense monitor (Respironics Minimitter, Bend, OR, USA) |
| Palmer, Clementi [61] | 75 GAD, 38 TD | Educational and Community-Based | 49% GAD, 47% TD | 6-12 | Sleep | Actigraphy device (Micro MotionLogger Actigraph Sleep Watches (Ambulatory  Monitoring, Inc., Ardsley, NY)) |
| Rolling, Rabot [62] | 11 PTSD, 11 controls with sleep disorder | Clinical and Medical Institution | 27% PTSD | 3-17 | Sleep | Actigraphy device (Motionwatch®, Cambridge Neurotechnology, UK; software (MotionWare 1.2.47) |
| Strumberger, Häberling [63] | 29 MDD, 29 TD | Clinical and Medical Institution | 21% MDD, 28% TD | 14,9 (1,81), 15,2 (1,74) | Sleep | Actigraphy device (ActTrust, Condor Instruments Ltda, SP, Brazil) |
| Aronen, Lampenius [64] | 30 ODD/CD, 30 TD | Clinical and Medical Institutions | 90% CD/ODD, 90% TD | 7-12 | Sleep | Actigraphy device (MicroMini– Motionlogger®, Ambulatory Monitoring, Inc., Ardsley, NY) |
| Bélanger, Desrosiers and Bernier [65] | 82 TD | Educational and Community-Based | 52% TD | 2-3 | Sleep | Actigraphy device (Mini-Mitter Actiwatch ; Respironics, Murrysville, PA) |
| Romanowicz, Croarkin [66] | 10 children with disruptive behavioural disorders (DBD) | Clinical and Medical Institution | 50% DBD | 7-10 | Sleep | Garmin vivosmart4 smartwatches |
| Stone, Cuellar [67] | 49 children with externalising behavioural symptoms | Clinical and Medical Institution | 44% children with externalising behavioural symptoms | 13 | Sleep | Actigraphy device (Octagonal Basics; AMI, Ardsley, NY) |
| Van Dyk, Thompson and Nelson [68] | 25 children with externalising behavioural symptoms | Clinical and Medical Institutions | 64% children with externalising behavioural symptoms | 6-11 | Sleep | ActiGraphTM |
| Wong, Brower [69] | 77 children of alcoholics, 38 TD | Clinical and medical institutions; Education and community based | 44 total | 8-12 | Sleep | Actigraphy device (Actiwatch-LTM, Mini-Mitter; Phillips Respironics,  Bend, OR) |
| Alban, Alhaddad [70] | 5 ASD | Clinical and Medical Institutions | 100% ASD | 7-10 | ANS | Empatica E4 wristbands, Empatica Inc. |
| Ali, Shah and Hughes [71] | 9 ASD | Not provided | 67% ASD | 8-11 | ANS | Samsung Galaxy 3 smartwatch |
| Anandhi, Jerritta [72] | 10 ASD, 10 TD | Educational and Community-Based | 70% ASD, 50% TD | 5-11 | ANS | Wearable Heart Wear ECG device |
| Bagirathan, Selvaraj [73] | 6 ASD, 6 TD | Educational and Community-Based | Not provided | 7-11 | ANS | Wearable Shimmer3 ECG device |
| Baker, Fenning [74] | 40 ASD | Educational and Community-Based | 80% ASD | 4-11 | ANS | Watch-like wireless Affectiva Q-Sensors (Picard et al., 2015) |
| Billeci, Tonacci [75] | 5 ASD | Not provided | 100% ASD | 6-8 | ANS | ECG chest belt (Solar et al., 2012)(based on the Shimmer R (Burns et al., 2010)) |
| Billeci, Tonacci [76] | 20 ASD, 20 TD | Clinical and Medical Institutions | 70% ASD, 75% TD | 1;6-3 | ANS | Electrocardiography (ECG) (CE certified Shimmer R platform (Burns et al., 2010)) |
| Costescu, Șogor [77] | 3 ASD | Not provided | 0% ASD | 3-5 | ANS | Xiaomi Mi Band 4 Fitness Bracelet |
| Fioriello, Maugeri [78] | 12 ASD | Clinical and Medical Institutions | 83% ASD | 3,6 (0,72), 3,5 (0,65) | ANS | Prototype wearable belt system with highlighted the 3-lead electrocardiograph |
| Goodwin, Mazefsky [79] | 20 ASD | Clinical and Medical database | 75% ASD | 6-17 | ANS | Empatica E4 wristbands, Empatica Inc. |
| Greenlee, Lorang [80] | 60 ASD | Not provided | 82% ASD | 5-18 | ANS | Empatica E4 wristbands, Empatica Inc. |
| Imbiriba, Demirkaya [81] | 70 ASD | Clinical and Medical Institutions | 89% ASD | 5-19 | ANS | Empatica E4 wristbands, Empatica Inc. |
| Krupa, Anantharam [82] | 10 ASD, 10 TD | Clinical and Medical Institutions | Not provided | 3-12 | ANS | GSR- and pulse sensors, an Arduino Pro Mini (3.3 V) Microcontroller |
| Kushki, Khan [83] | 24 ASD | Not provided | 71% ASD | 11,7 (2,8) | ANS | Wearable sensor from Shimmer research with Three-lead electrocardiogram (ECG) |
| Nuske, Finkel [84] | 13 ASD | Databased | 77% ASD | 2-4 | ANS | Empatica E4 wristbands, Empatica Inc. |
| Nuske, Goodwin [85] | 32 ASD, 23 TD | Not provided | 77% ASD, 74% TD | 9,7 (1,47), 9,6 (1,23) | ANS | Chest straps and wristbands: Polar H7 ECG chest-strap, Mio Fuse PPG wristband, and PulseOn PPG wristband |
| Lønfeldt, Olesen [86] | 9 OCD | Clinical and Medical Institutions | 44% OCD | 10-16 | ANS | Empatica E4 wristbands, Empatica Inc. |
| Schuurmans, De Looff [87] | 15 PTSD | Clinical and Medical Institution | 60% PTSD | 14,46 (2,40) | ANS | Empatica E4 wristbands, Empatica Inc. |
| Naim, Goodwin [88] | 16 ADHD, 26 internalizing, 9 TD | Educational and Community-Based | 63% total | 8,12-17,76 | ANS | Empatica E4 wristbands, Empatica Inc. |
| Chu, Lu [89] | 49 ADHD, 14 TD | Educational and Community-Based | 82% ADHD, 36% TD | 6-12 | Brain activity and Motion | Mobile EEG device (MindSet; NeuroSky); Actigraphy device (MotionWatch 8; CamNtech) |
| Dekkers, Rapport [90] | 36 ADHD, 24 TD | Educational and Community-Based | 92% total | 8-12 | Motion and activity | Actigraphy device (MicroMini– Motionlogger®, Ambulatory Monitoring, 2004) |
| Hartanto, Krafft [91] | 26 ADHD, 18 TD | Not provided | 54% ADHD, 28% TD | 10-17 | Motion and activity | Actigraphy device, Motionlogger® (Ambulatory Monitoring, Inc., Ardsley, NY, USA) |
| Hudec, Alderson [92] | 19 ADHD, 18 TD | Clinical and Medical Institution and Education and Community based | 100% total | 8-12 | Motion and activity | Actigraphy device (MicroMini Motionlogger1 (Ambulatory Monitoring Inc., 2010)) |
| Patros, Alderson [93] | 15 ADHD, 17 TD | Clinical and Medical Institution | 100% ADHD, 100% TD | 8-12 | Motion and activity | Actigraphy device, MicroMini Motionlogger Actigraphs (Ambulatory Monitoring, 2010) |
| McGinnis, McGinnis [94] | 21 internalizing, 40 TD | Educational and Community-Based | 43% total | 3-7 | Motion and activity | Belt-worn IMU (3-Space Sensor, YEI Technology, Portsmouth, OH, USA) |
| Merikanto, Partonen [95] | 8 depression, 9 TD | Clinical and Medical Institution | 100% depression, 100% TD | 16,0 (1.1), 16,0 (0,7) | Motion and activity | Actigraphy device (Actiwatch-Plus®, Cambridge Neurotechnology Ltd, Cambridge, UK) |
| Cantin-Garside, Kong [96] | 11 ASD | Clinical and Medical Institutions & Education and Community-based | 64% ASD | 5-14 | Motion and activity | Actigraphy device (Tri-axial accelerometers; ActiGraph GT9X) |
| McCabe, Johnstone [97] | 112 ADHD, 255 TD | Clinical and Medical Institution | 84% ADHD, 54% TD | 7-12 | Brain activity | Single-channel, dry-sensor, portable device (Mindwave Mobile II, Neurosky, San Jose, California) |
| Ortuño-Miró, Molina-Rodríguez [98] | 15 ADHD, 15 TD | Educational and Community-Based | 100% ADHD, 100% TD | 10-13 | Brain activity | NIRS device (Tehia,Newmanbrain,S.L.,Elche,Spain), |
| Barreto, Curtin [99] | 12 ASD, 16 TD | Educational and Community-Based | 75% ASD, 38% TD | 3,89 (1,26), 2,88 (0,63) | Brain activity | Continuous wave fNIRS Imager Model 2000S  (fNIR Devices, LLC, Potomac, MD, USA) |
| Clin, Miller and Kissine [100] | 18 ASD, 36 TD | Educational and Community-Based | 83% ASD, 83% TD | 6-9 | Eye gaze | Head-mounted eye-tracker |
